# Understanding the mechanisms behind the sexualized-body inversion hypothesis: The role of asymmetry and attention biases

**DOI:** 10.1371/journal.pone.0193944

**Published:** 2018-04-05

**Authors:** Carlotta Cogoni, Andrea Carnaghi, Aleksandra Mitrovic, Helmut Leder, Carlo Fantoni, Giorgia Silani

**Affiliations:** 1 Department of Psychology and Cognitive Science, University of Trento, Rovereto, Italy; 2 Scuola Internazionale Superiore di Studi Avanzati, Neuroscience Sector, Trieste, Italy; 3 Dipartimento di Scienze Della Vita, Università degli Studi Di Trieste, Trieste, Italy; 4 Department of Basic Psychological Research and Research Methods, University of Vienna, Vienna, Austria; 5 Department of Applied Psychology: Health, Development, Enhancement and Intervention, University of Vienna, Vienna, Austria; University of Bologna, ITALY

## Abstract

A controversial hypothesis, named the Sexualized Body Inversion Hypothesis (SBIH), claims similar visual processing of sexually objectified women (i.e., with a focus on the sexual body parts) and inanimate objects as indicated by an absence of the inversion effect for both type of stimuli. The current study aims at shedding light into the mechanisms behind the SBIH in a series of 4 experiments. Using a modified version of Bernard et al.´s (2012) visual-matching task, first we tested the core assumption of the SBIH, namely that a similar processing style occurs for sexualized human bodies and objects. In Experiments 1 and 2 a non-sexualized (personalized) condition plus two object-control conditions (mannequins, and houses) were included in the experimental design. Results showed an *inversion effect* for images of personalized women and mannequins, but not for sexualized women and houses. Second, we explored whether this effect was driven by differences in stimulus asymmetry, by testing the *mediating* and *moderating* role of this visual feature. In Experiment 3, we provided the first evidence that not only the sexual attributes of the images but also additional perceptual features of the stimuli, such as their asymmetry, played a *moderating* role in shaping the inversion effect. Lastly, we investigated the strategy adopted in the visual-matching task by tracking eye movements of the participants. Results of Experiment 4 suggest an association between a specific pattern of visual exploration of the images and the presence of the inversion effect. Findings are discussed with respect to the literature on sexual objectification.

## Introduction

The idea that women and men can be treated like objects, as a function of their sexual attributes, has increasingly attracted attention of the public at large[1], especially because of the important implications at a societal level [2, 3]. Bartky [4] defined this phenomenon as sexual objectification: a condition in which the individual´s sexual parts or sexual functions are separated out from the person, reduced to the status of mere instruments, as if they were able of representing her/him. In the last decades, the scientific community has begun to investigate the cognitive mechanisms and consequences associated with such a phenomenon. It has been observed that when a person (especially women) has been sexually objectified (sexualized from now on), she is likely to be perceived as deprived of her mind, moral status [5] and agency [6], which are core characteristics that distinguish humans from animals and nonliving things [7]. Interestingly not only high-level cognitive processes (such as mind attribution and empathy [8]) seem to be modulated by the perceived sexualization of the target, but also low-level cognitive processes (such as perceptual recognition). For instance, Gervais and colleagues [9], showed part-to-whole effects with only women´s sexual body parts being recognized equally well regardless of whether they were presented in the context of the entire body or in isolation, while men’s sexual body parts were recognized better when they were presented in the context of the entire body rather than in isolation. The authors interpreted this pattern of results in the context of the sexual body part recognition bias hypothesis, on the basis of which different cognitive processing styles would be adopted for women vs. men: namely a local, part-based (or analytical) vs. global (holistic or configural) visual processing, respectively associated with object and human body recognition [10]. In a similar vein, the seminal work of Bernard and colleagues [11–13] measured the size of the inversion effect (lower performance when stimuli are presented in the unusual upside-down orientation, an indicator of configural processing [10, 14–19], but see also [20] for a different interpretation of the processes behind the inversion effect), in order to ascertain the processing style associated with the perception of sexualized targets. Bernard and colleagues found that sexualized women, but not men, both portrayed in swimsuits, were recognized equally well when presented in the upright and the inverted orientation. Bernard and colleagues put forward that sexualized women but not men were processed in an object-like fashion, which is the core claim of the so-called sexualized-body inversion hypothesis (SBIH).

The work of Bernard and colleagues has opened a vibrant debate that is still ongoing today. The main criticisms directed to their findings point to the absence of control over confounding variables, which could have provided alternative explanations of their results [21, 22]. In particular, Tarr [21] suggested that non-social perceptual factors could account for the sexualized-body inversion effect (SBIE) observed by Bernard. Specifically, the perceptual properties of the stimuli, such as their structural complexity, distinctiveness and asymmetry, may have played a key role in shaping the effect. Another factor that could account for the observed results without advocating a difference in perceptual processing per se [23] is related to attention. Participants’ greater attention to female rather than male bodies could have resulted in a better recognition performance for inverted female images (note that this hypothesis was lately ruled out by Bernard [13] suggesting that longer reaction times, possibly indicating higher attention allocation, were associated with poorer performance). Lastly, a non-sexualized condition was not included in the study, preventing the authors from concluding that the sexualized nature of the stimulus accounted for the observed pattern of results.

Following Tarr's critical view, Schmidt and colleagues [22] analyzed the original stimuli of Bernard and found higher asymmetry in the female pictures, compared to the male ones, and higher asymmetry in the inverted compared to the upright ones. When symmetry was controlled, by using a newly developed dataset of stimuli, Schmidt and colleagues did not find significant differences in the size of the inversion effect between men and women pictures, and between a sexualized and less sexualized condition. Few recent studies tips the balance again in favor of the SBIH, revealing that symmetry (or other low level visual features) is not the fundamental mechanism that drives the SBIE [12, 24]. By using the same stimuli in different conditions, both Bernard and colleagues as well as Civile and colleagues, replicated the initial findings of Bernard et al. [11]. These studies also showed that the SBIE is sensitive to contextual factors, such as the perceived humanness of the target [12] and the power of the perceiver [24, 25].

In spite of the increasing evidence in favor of the SBIE, several questions remain unresolved, such as: 1) how do different levels of sexualization modulate the SBIE? 2) How do symmetry and level of sexualization interact in facilitating the SBIE? And 3) can the SBIE be explained by attention biases that are reflected, for example, in focusing on different body-parts? Furthermore, differences in the occurrence of the inversion effect in sexualized targets has never been related to its occurrence in the processing of real objects, making the claim that sexually objectified women are processed like objects more hypothetical, rather than being formally tested.

In the present work, we aimed to shed new light on the current debate and address three different, albeit related points. First, by including two object-control conditions (namely objects with a human-body shape, such as mannequins, and houses), we tested the core assumption of the SBIH that puts forward a similar cognitive processing style for sexualized female targets and objects. Second, to ascertain the role of asymmetry in the occurrence of the SBIE, we tested the mediating and moderating role of this feature in determining (or not) the SBIE in a sexualized and less-sexualized (personalized from now on) condition. Third, for the first time in the domain of the SBIH, we investigate the recognition strategy adopted in the image recognition task by tracking the eye movements during the performance, thus characterizing the relationship between the SBIE and the presence of attention biases.

### Testing the core assumption of the SBIH

The core assumption of the SBIH is that sexualized women and objects are processed in a similar analytical manner, as indicated by an absence of the inversion effect. However, it is known that the inversion effect can occur with objects [21] with a human body-like shape [26–28]. This makes the aforementioned statement “processed like objects” possibly misleading. To date, only one study has introduced a real-object control condition to test differences in the neural processing of inverted objects and sexualized women [29]. However, no direct comparison has been carried out with the most-frequently used paradigm to test the SBIH [11]. To address this issue, we assessed the extent to which analytical processing varied with different types of objects and different types of women. In doing so we aim to test the hypothesis that it is not the object-like nature of the stimuli but rather the level of sexualization that accounts for the presence or absence of the inversion effect.

In Experiments 1 and 2, two control conditions were employed, consisting of houses and human body-like objects (mannequins). We selected these two kinds of objects for three distinct, albeit related reasons. We included houses since they: (1) have been extensively used in previous research when comparing object to human body recognition [26, 30–32]; (2) generally do not show inversion effects [10]; and (3) are shapes with minimal asymmetry along the horizontal axis. We included mannequins with a woman-like shape to investigate how objects that have a silhouette similar to women are processed. Interestingly, it has been already shown [33] that the more an entity has human-like features, the more it is humanized by the perceiver (i.e., a model of the mind is constructed); at the same time, mannequins are likely to be perceived as less sexy compared to real women. Both features (i.e., the human body-like shape and the reduction of sexual attributes) may have an impact on the inversion effect.

Furthermore, a sexualized and personalized condition were used, by selecting pictures that represent women in real-life clothing, with the intent to assess the occurrence of the SBIE with every-day images. The same women were shown either wearing a swimsuit (sexualized condition) or casual clothes (personalized condition). The operationalization of a sexualized and personalized condition was based on the current literature [9, 34, 35], which considered the amount of uncovered skin (and not the fact of being naked per se) as being the driving factor for the emergency of the perceived sexualization of the target.

In line with the results of Bernard and colleagues [11, 12] and Schmidt and Kistemaker [22], in Experiments 1 and 2 we expected to find no difference in terms of accuracy when participants have to recognize both sexualized women and houses in the upright or in the inverted orientation. In contrast, participants were expected to be less accurate when recognizing personalized women in the inverted than in the upright orientation. As for mannequins, if their human body-like non-sexual features trigger a humanized representation, then one would expect participants to adopt a configural processing style, with better recognition in the upright than in the inverted orientation.

#### On the mediating and moderating role of stimulus asymmetry in the SBIE

As highlighted by Tarr [21], properties of visual stimuli such as asymmetry can play an important role in modulating the inversion effect. In human silhouettes, asymmetry between body points can be easily quantified by calculating the angle formed by the connection of the right and left point of interest (i.e. shoulder), in conjunction with the horizontal axis [22]: the wider the angle, the higher the asymmetry of that point. This analysis is particularly relevant because asymmetry can be either a moderator or a mediator variable of the SBIE. According to Baron and Kenny [36], a mediator variable represents the ‘generative mechanism’ by which an independent variable affects the dependent variable. In other words, the independent variable is able to impact on the dependent variable because it alters an intervening, third variable. Recasting this definition within the SBIH research, the difference in terms of asymmetry (i.e., potential mediator) between the stimulus pictures, for example sexualized men vs. women (i.e., independent variable), might account for the different impact of these stimuli on the inversion effect (i.e., dependent variable). On the other hand, a moderator variable refers to the division of an independent variable into subgroups that define its domains of maximal effectiveness on the dependent variable. So, the moderator variable shapes the direction of the effects of the independent variable on the dependent variable. Recasting this definition within the SBIH research, the experimental manipulation of asymmetry requires some stimuli with high and others with low asymmetry (i.e., potential moderator). This will allow us to specify the conditions under which the stimulus pictures (i.e., independent variable) might (or might not) exert a different impact on the accuracy (i.e., dependent variable) in the visual matching task.

Based on this rationale, in Experiments 1 and 2 we relied on the experimental protocol outlined by Bernard et al. [11], and used a new but comparable set of visual stimuli. More importantly, we let the level of asymmetry of the stimuli co-vary with the level of sexualization of these stimuli, namely the higher the sexualization of the stimuli, the higher the asymmetry of the stimuli in question. We considered the house stimuli as a relevant baseline condition with zero sexualization and almost zero asymmetry (houses are characterized by having straight lines with walls, windows and doors always parallel to the horizontal line). In doing so, we tested whether asymmetry *mediates* the SBIE, thus ascertaining whether the original findings reported by Bernard et al. [11] were indeed driven by a perceptual artifact. It should be noticed that, albeit Schmidt and Kistemaker [22] assessed the level of stimulus asymmetry in the original dataset used by Bernard, no mediational analysis has been carried out to directly test their claim.

In Experiment 3 we systematically varied the stimulus asymmetry, thus addressing whether it can moderate the occurrence of the inversion effect. Indeed, when highly compared to weakly asymmetric stimuli are used, accuracy in the visual matching task may be facilitated (‘the more asymmetric the stimuli, the easier the task’; Schmidt and Kistemaker [22] p.78). Finally, in Experiment 4, we used stimuli with different levels of sexualization and gender but equal in asymmetry, allowing us to extend the findings to both male and female pictures. Two alternative hypotheses can be put forward. First, if the asymmetry plays a mediating role, upright and inverted pictures should be better recognized when pictures are highly asymmetric compared to weakly asymmetric, regardless of the level of sexualization. This pattern of results would support the claim that the SBIE is mostly driven by visual artifacts. In contrast, if asymmetry moderates the recognition of upright and inverted pictures differently for more and less sexualized pictures, then the claim that the SBIE is driven by visual artifacts should be dismissed. In particular, the finding that upright and inverted highly asymmetric stimuli are processed the same, independently of the level of sexualization, would support the hypothesis that asymmetry facilitates recognition. By contrast, if an inversion effect occurs for weakly asymmetric personalized, but not sexualized stimuli, then asymmetry alone cannot explain the occurrence of the SBIE.

Addressing the mediating and/or the moderating role of the stimulus asymmetry within the SBIE would clarify *why* the observed effects occur (mediation) and *when* the effects hold (moderation). The combined findings on the mediating and moderating role of the asymmetry in the visual matching task would allow for either corroborating or dismissing the SBIH.

#### The role of the focus of attention in shaping the SBIE

The SBIH claims different recognition strategies (analytical and configural) adopted for each class of stimuli (women vs. men). Another non-social (possible) explanation of this effect could be linked to a different attentional focus during the visual exploration of the stimuli. It has already been shown that participants tend to fixate the chest and pelvic region longer compared to the face region when scanning pictures depicting naked compared to dressed people [37]. Similar results have been found by Gervais et al. [38], who reported that participants focused longer on women’s chests and waists, than on faces, when asked to evaluate the attractiveness of pictures of models. Moreover, this effect was particularly pronounced for women with more (vs. average and less) ideal body shape (pronounced breast and lower waist-to-hip ratio). However, how the SBIE is related to differences in the visual exploration of the stimuli (i.e., spatial attention) during the matching task is still an open question. Preliminary evidence in favor of differing patterns of visual exploration as a function of the type of stimuli comes from the same work by Bernard and colleagues [12]. The authors in fact observed that the SBIE is sensitive to a change in saliency of the sexual attributes, possibly indicating a shift of attention to other body parts (i.e., face). Recording eye movements with the Eye-tracker allowed us to directly test this hypothesis.

Therefore, in an exploratory Experiment 4, participants’ eye movements were recorded with an eye-tracker device during the visual matching task. Importantly, in order to keep the paradigm as close as possible to the original work of Bernard and colleagues [11], the target images were presented for a short duration (250ms). The decision not to change the presentation duration was also based on previous literature indicating the occurrence of saccades within very short time periods [39, 40]. Nevertheless, given the exploratory nature of Experiment 4 is important to consider these restrictions on the generalizability of the findings. We predicted that the visual exploration of personalized images would be more focused on the face as compared to the chest and the pelvic region. This focus on the face may trigger a configural recognition style leading to the emergence of the inversion effect when presented with inverted stimuli. On the other hand, we predicted higher focus on the chest and pelvic region during the exploration of sexualized images, which were also expected to be equally well recognized in both orientations (absence of the inversion effect).

### Experiment 1–2

Experiments 1 and 2 were performed with the same stimuli and procedure but in a between and within–subject fashion, respectively. Given the similarity of the results, the full descriptions and statistics of Experiment 1 is reported in S1 File. In the following paragraphs Experiment 2 is described.

## Method

### Participants

Eighty healthy students (*N* = 40 men and *N* = 40 women; age *M* = 23.06, *SD* = 3.23 years) took part in the present study in exchange for monetary reward. The study was conducted at the International School for Advances Studies (SISSA-ISAS), Trieste, Italy. All participants gave written informed consent before participating in the study, which was approved by the SISSA ethical committee and were treated in accordance with the Declaration of Helsinki. Participants were naïve to the aim of the study and did not participate in similar studies before. Sample size calculation for Experiment 1 (minimum N = 84) was based on power analyses for a medium effect size (f = .44) (e.g., [11]) and standard parameters of α = .05 and 1 –b = .80 [41]. Sample size calculation for Experiments 2–4 (minimum N = 36), was based on power analyses for a medium effect size (f = .2) (e.g., [11]) and standard parameters of α = .05 and 1 –b = .80 [41].

### Procedure

Participants took part in a picture recognition task with a similar procedure as in Bernard at al. [11], but with a novel set of pictures. Each participant saw a total of 96 pictures: 24 personalized women, 24 sexualized women, 24 mannequins and 24 pictures of houses.

In order to avoid the repetition of the same stimulus and therefore a facilitation effect, each participant was randomly presented (within each condition: personalized, sexualized, mannequins and houses) with half of the stimuli in the upright orientation and half in the inverted orientation (on the x axis: top-down). For each trial, a probe picture appeared at the center of the computer screen for 250 *ms*, followed by a blank screen for 1000 *ms*. Immediately after, participants were presented with two pictures: one on the left and one on the right side of the center of the screen. One picture was the exact copy of the probe while the other was its left-right mirrored version. Participants were requested to indicate which of the two pictures (the one on the left or the one on the right) matched with the probe (Figure B in S1 File), by pressing a key on the keyboard with the same position along the horizontal of the matched picture (i.e., the “L” if right, vs. “A” if left). Pictures were presented on a computer screen using Cogent Toolbox (http://www.vislab.ucl.ac.uk/cogent.php), running on Matlab 2011a. Before starting the experiment, participants completed four practice trials, in order to familiarize with the task. The individual accuracy scores were recorded and analyzed for all the conditions.

The analyses of accuracy scores were performed using RStudio software (version 3.3.2). As for the analyses on the mediating role of asymmetry on the inversion effect, the bootstrap Lavaan R software [42] was used with 1000 iterations for the implementation of a Structural Equation model without latent variables. The analyses on asymmetry and pretest evaluations were instead performed using IBM statistics software SPSS, version 21.

### Stimuli

**Fig 1:**
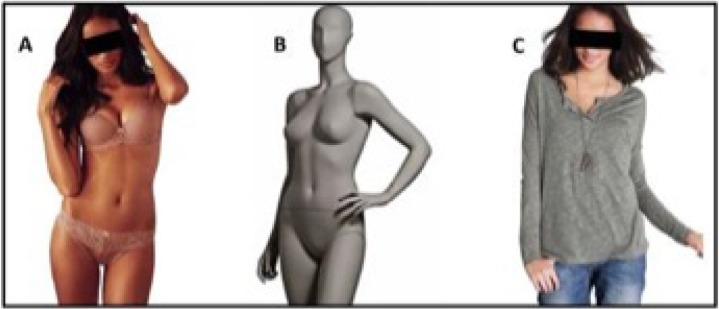
Exemplar target stimuli. Picture of a sexualized woman (A), a mannequin (B), a personalized woman (C). All stimuli were presented without covering black bars.

The experimental stimulus set consisted of 96 pictures in total (Fig 1): 24 pictures of women wearing a swimsuit or underwear, with 86% of their body left uncovered (sexualized condition), 24 pictures of women wearing casual/classic clothes, with 28% of their body left uncovered (personalized condition), 24 pictures of woman-shape mannequins without clothes (mannequin condition) and 24 pictures of houses (house condition) (see Fig 1 for an exemplar of each stimulus´ category and Figure A in S1 File for an exemplary house stimulus).

Stimuli were collected from free sources from the web. In particular, for the pictures of the women, pictures of the same model portrayed in the sexualized as well as in the personalized condition were selected, thus preserving identity across conditions. Pictures of houses were chosen from the “Pasadena houses dataset” [43].

Pictures of women and mannequins were depicted from head to knee, in a standing position, with eyes/face focused on the camera. All pictures were modified to have a white background, the same luminance and the dimension of 397 x 576 Pixels. Stimuli were shown on a Samsung B1940 display monitor at a resolution of 1280x1024 pixels and with 60Hz of refresh rate. The viewing distance was 65 cm with the head aligned with the monitor.

In order to confirm that the selected sexualized women were perceived as more attractive and sexy than the personalized women, and to gather information on the way mannequins were perceived on these dimensions, a pretest was conducted on an independent pool of 30 participants issued from the same population of the experimental sample. They rated the personalized women, sexualized women and mannequins on a 6-point scale, ranging from 1 (= not at all) to 6 (= completely) on the perceived level of attractiveness, sexiness, intelligence and familiarity. As expected, sexualized women were rated as sexier and more attractive than the personalized ones and the mannequins; the personalized women were rated as more attractive than the mannequins. Also, the personalized women were rated as more intelligent than the sexualized ones and the mannequins, and the sexualized women as more intelligent than the mannequins. Finally, the personalized women were rated as more familiar than the mannequins and the sexualized ones; all the other comparisons did not reach significance level (see S1 File for the full statistic).

In addition, following the procedure introduced by Schmidt and Kistemaker [22], an asymmetry index was calculated for each human body-like picture.

### Analysis of the stimuli asymmetry

Following the procedure of Schmidt and Kistemaker [22], we focused on the angles of four body-axes measured for the three conditions (personalized, sexualized and mannequin): shoulders, elbows, hands and hips. Schmidt and Kistemaker [22] also took into consideration the angle of the eyes but this measure was not applicable for all three conditions because pictures of mannequins did not have eyes. Therefore, this parameter was not considered (note that an independent sample *t*-test revealed no differences in the eyes´ angles between the pictures of sexualized (*M* = 6.85, *SD* = 9.55) and personalized women (*M* = 9.80, *SD* = 11.18), *t* (46) = -.98, *p* = .33, *d* = .28.

Taking into consideration the Average Axes values (mean of the four different axes), a one-way ANOVA was conducted. Results showed a significant difference between conditions (sexualized, personalized and mannequin) *F* (2, 69) = 15.84, *p* < .001, *η*_*p*_^*2*^ = .32. Post-hoc analyses revealed that sexualized women (*M* = 22.45, *SD* = 10.02) were more asymmetric than personalized women (*M* = 14.91, *SD* = 9.32), *t* (46) = 2.70, *p* = .01, *d* = .78; and mannequins (*M* = 8.96, *SD* = 4.47), *t* (46) = 6.02, *p* < .001, *d* = 1.74 and that personalized women were more asymmetric than mannequins, *t* (46) = 2.81, *p* = .007 *d =* .81 (see Table C in S1 File for the separate axes values).

Note that a separate analysis for the pictures presented in the upright and inverted orientations was not necessary given that every picture was randomly assigned to both conditions.

## Results

### Accuracy

Following Knoblauch and Maloney [44], individual standardized values of matching accuracy were analyzed encoding individual responses as a binary variable in terms of correct (1)—incorrect (0) matchings and sending the whole pattern of binary responses to a generalized linear mixed effect model (*glmer*) with a probit link function. The *glmer* was performed using the *mixed* function of the package for Analysis of Factorial Experiments (afex, v.0.13–145), running on lme4 (v.1.1–7). To avoid derivative calculation, an optimizer (bobyqa) was chosen. Our *glmer* model included an independent random intercept for every subject and condition (houses, sexualized, personalized, mannequin), orientation (upright, inverted) and gender of the participant (male, female) as fixed effects. In particular, participants were treated as random effect to control for the individual variability of matching performance as signaled by individual accuracy (computed as the percentage of corrected answers).

In order to optimize the statistical validity of our dataset we decided to exclude from the analysis participants collecting a performance below the chance level (i.e., 75% correct) in more than 37.5% of tested experimental conditions (n = 8). This results in the exclusion of 8 out of the 80 participants. After the application of this exclusion criteria, we performed an outlier analysis on individual pattern of correct/ incorrect responses: trials in which any one of the considered individual binary response deviated more than 4 SD from the individual best fitting *glmer* model, including all interaction terms as fixed factors, were removed from the analyses. This analysis lead to the exclusion of 3 trials out of the remaining 6912.

**Fig 2:**
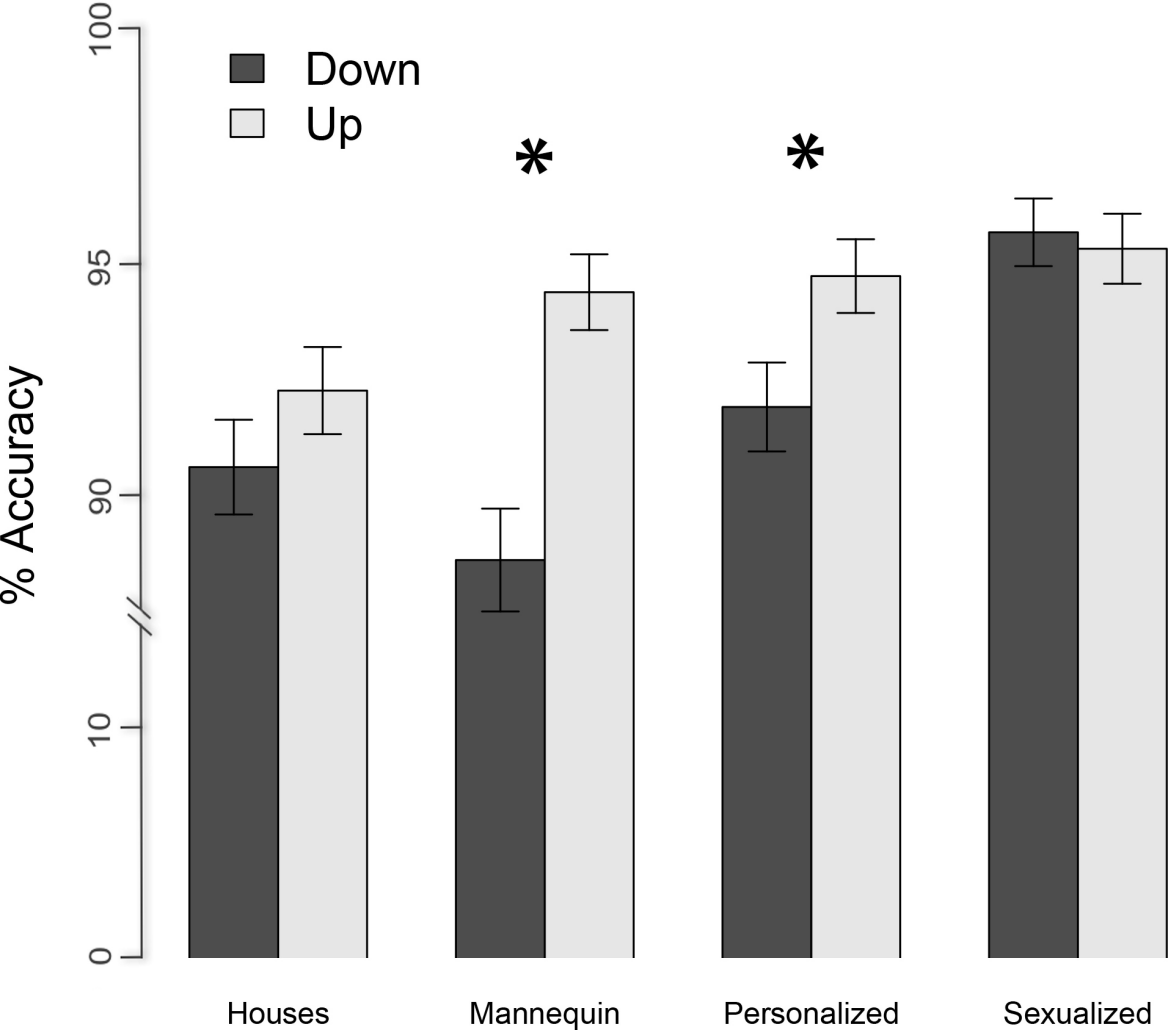
Accuracy. Mean and SE values of the proportion of accuracy as a function of the condition (personalized, sexualized, mannequin and houses). As by the legend, the color encodes the orientation (up for upright and down for inverted). The asterisk indicates the presence of the inversion effect. To be noted that in order to simplify Figures and Tables in all the manuscript the upright orientation has been indicated by the word “up” while the inverted orientation has been indicated by the word “down”.

Results revealed a significant main effect of orientation, *F* (1, 6908) = 14.26, *p* < .001, which was moderated by the condition, *F* (3, 6906) = 3.26, *p* = .02. In order to clarify the origin of such an interaction we looked at the effect of orientation on the accuracy scores separately for each condition and run a *glmer* model containing only the orientation as the main effect and the participants as random effects, separately for each condition subset. Analyses indicated that the pictures were better recognized in the upright than the inverted orientation in the personalized (*glmer* estimated accuracy for upright vs. inverted = .95 ± .02 vs. .92 ± .02; *F* (1, 1726) = 5.16, *p* = .02) and in the mannequin (*glmer* estimated accuracy for upright vs. inverted = .94 ± .02 vs. .89 ± .02; *F* (1, 1726) = 17.26, *p* < .001) condition but not in the sexualized (*glmer* estimated accuracy for upright vs. inverted = .96 ± .02 vs. .96 ± .02; *F* (1, 1725) = .09, *p* = .76) and in the house condition (*glmer* estimated accuracy for upright vs. inverted = .92 ± .02 vs. .91 ± .02; *F* (1, 1726) = 1.55 *p* = .21). All the other effects and interactions did not reach significance *p* > .12 (See Fig 2).

### Mediation analysis on the asymmetry index

In order to corroborate the results from the *glmer* and to test whether the SBIE was mainly driven by the different asymmetry of stimuli belonging to different conditions (house, mannequin, sexualized or personalized) a causal mediation analysis was performed. In order to do that, we analyzed how the inversion effect displayed by each image (calculated as the difference between the proportion of correct responses in upright vs. inverted orientation), was accounted by the condition and/or by the level of asymmetry. The analysis was thus performed on 96 inversion values (resulting from the combination of 4 conditions × 24 images) contrasting linear models (*lm*) allowing to establish the mutual relationship between the causal variable (i.e., condition), the potential mediator (i.e., asymmetry), and the outcome (i.e., inversion effect).

Such analysis thus allowed us to investigate to what extent the way in which condition and orientation interact to systematically affect the performance (as revealed by the *glmer* analysis) can be accounted for their effect on asymmetry as a mediator, which in turn affects the inversion effect.

Through contrasting multiple *lm* models we thus inferred: 1) the Total Effect (*c* path) of the condition on the inversion effect, 2) whether the condition contributes to the variance of the asymmetry as a mediator (*a* path), 3) the Indirect Effect (*b* path) examining to what extent the mediator contributes to the variance of the inversion effect, 4) the Direct Effect (*c’* path) providing a measure of whether the condition continued to predict the inversion effect with the mediators in the model. Coefficients associated to meaningful paths have been estimated using a Structural Equation Model (SEM) without latent variables and with condition as predictor, asymmetry as mediator, and inversion effect as outcome.

The analysis revealed a good model fit [45] with the Comparative Fit Index (CFI) always larger than 0.95 (CFI = 0.99, 1st to 3rd quartile 0.989 to 0.999) and the Standardized Root Mean-square Residual (SRMR) always smaller than 0.08 (SRMR = 0.0321, 1st to 3rd quartile 0.0115 to 0.0474). The model was characterized by a significant Total Effect (*c* path) (*r*^*2*^ = 0.10, *F*(3, 92) = 3.42, *p* = 0.02) with a minimal though not different from zero inversion effect for the house condition (*lm* estimated inversion effect = 0.018 ± 0.0139, *t* = 1.313, *df* = 92, *p* = 0.19), which was smaller than the inversion effect found in the mannequin (*lm* estimated inversion effect difference from the house inversion effect = 0.0415 ± 0.019, *t* = 2.109, *df* = 92, *p =* 0.037) but not from the one found in the personalized women condition (*lm* estimated inversion effect difference from the house inversion effect = 0.009 ± 0.01967, *t* = 0.453, *df* = 92, *p* = 0.65) and in the sexualized women condition (*lm* estimated inversion effect difference from the house inversion effect = - 0.020 ± 0.01967, *t* = -1.032, *df* = 92, *p* = 0.30). Notably the magnitude of the Total Effect calculated on the inversion effect values was of about the same statistical entity of the condition × orientation interaction revealed by the *glm* analysis on the individual patterns of correct responses. This demonstrated that the 96 inversion effect values used here to infer the mediating role of asymmetry on the performance provided a reliable synthetic measure of individual performance.

Furthermore, as the conditions significantly contributed to the variance of the inversion effect it also contributed to the variance of the asymmetry (SEM estimated coefficient = 5.822 ± 0.794; *z* = 7.331, *p* < 0.001; *r*^*2*^ = 0.45, *F*(3, 92) = 25.4, *p* < 0.001), with the asymmetry of the mannequin (*lm* estimated asymmetry = 8.967 ± 2.079, *t* = 4.313, *df* = 92, *p* < 0.001) been lower, the one of the personalized women been intermediate (*lm* estimated asymmetry = 14.91 ± 2.079, *t* = 7.172, *df* = 92, *p* < 0.001), and of the sexualized women (*lm* estimated asymmetry = 22.45 ± 2.079, *t* = 10.79, *df* = 92, *p* < 0.001), been maximal relative to the asymmetry imposed in the present analysis to houses as a baseline (i.e., 0). This result corroborated the condition by asymmetry co-variation revealed by the preliminary analysis of the asymmetry of the stimuli used in our dataset.

Moreover, the relation between asymmetry and inversion effect (*b* path) resulted to be not statistically significant (SEM estimated coefficient = 0.001 ± 0.001, *z* = -1.637, *p* = 0.102) both when considered directly (*r*^*2*^ = 0.027, *F*(1, 94) = 2.65, *p* = 0.107; *lm* estimated coefficient = -0.0001 ± 0.0006, *t* = -1.629, *df* = 92, *p* = 0.106) following James and Brett (1984), and indirectly (*lm* estimated coefficient = -0.001, ± 0.0009, *t* = -1.04, *df* = 91, *p* = 0.29) [36] as controlling for the effect of the condition as a causal variable. However, the direct association between condition and inversion effect was not significantly affected by the addition of the asymmetry as a mediator (c’ path) (*F*(1, 94) = 3.42, *p* = 0.02). This was further corroborated by the fact that a significant loss in the fit was found when contrasting an *lm* with asymmetry as the only predictor of inversion effect with an *lm* including both asymmetry and condition (*F*(1, 94) = 2.85, *df* = 3, *p* = 0.04). The results of the mediation analysis thus provide no evidence that the asymmetry of images mediates the differential effect of inversion on matching performance observed among the different categories of images.

## Experiment 3

In order to formally test the moderating role of asymmetry in shaping the SBIE we run Experiment 3. Participants were engaged in the same task described in Experiments 1 and 2, with the exception that mannequins and houses were removed from the stimulus set. Importantly, and differently from Experiments 1 and 2, the asymmetry of the stimuli was experimentally manipulated, including half of the stimuli with low asymmetry and with high asymmetry in both personalized and sexualized conditions. This allowed us to directly test the moderating role of asymmetry in the emergence of the inversion effect. We expected the high asymmetrical images to be well recognizable and therefore that the inversion effect should not be influenced by the level of sexualization. On the other hand, we expected that for images that are difficult to be recognized, like the low asymmetrical ones, sexualization will play a role in shaping the inversion effect.

## Method

### Participants

Seventy-seven healthy students (*N* = 38 men and *N* = 39 women; age *M* = 22, *SD* = 2.83 years) took part in the present study in exchange for monetary reward. All participants gave written informed consent before participating in the study, which was approved by the SISSA ethical committee and were treated in accordance with the Declaration of Helsinki. Participants were naïve to the aim of the study and did not participate in similar studies before.

### Stimuli

Pictures used in Experiment 3 were collected from the same sources as in the other experiments. We took care of obtaining pictures of equally low asymmetry as well as equally high asymmetry for the sexualized and personalized stimuli. Concretely, each participant saw a total of 48 pictures: 24 sexualized women and 24 personalized women, each group consisted of 12 pictures with low asymmetry and 12 pictures with high asymmetry (see S1 File for the asymmetry indexes). The stimulus size, viewing conditions and apparatus were the same as in Experiment 1 and 2.

#### Analysis of asymmetry

A 2 condition (sexualized, personalized) x 2 asymmetry (high, low) univariate ANOVA was carried out on the Average Axes values (mean of the five different axes) as shown in Table D in S1 File. Results showed a main effect of the asymmetry, *F* (1, 44) = 516,912, *p* < .001, *η*_*p*_^*2*^ = .92, indicating that high asymmetrical pictures had more prominent axes than low asymmetrical ones. The other main effect and interaction did not approach the significance level *p* > .33.

## Results

### Accuracy

Individual accuracy in the matching task was analyzed with the same statistical technique described in Experiment 1 and 2. We used a *glmer* with an independent random intercept for every subject and with condition (sexualized, personalized), orientation (upright, inverted), asymmetry (high, low) and gender of the participant (male, female) as fixed factors.

In order to optimize the statistical validity of our dataset we decided to exclude from the analysis participants collecting a performance below the chance level (i.e., 75% correct) in more than 37.5% of tested experimental conditions (n = 8). This results in the exclusion of 8 out of the 77 participants. After the application of this exclusion criteria, we performed an outlier analysis on individual pattern of correct/ incorrect responses: trials in which any one of the considered individual binary response deviated more than 4 SD from the individual best fitting *glmer* model, including all interaction terms as fixed factors, were removed from the analyses. This analysis lead to the exclusion of 37 trials out of the remaining 3456.

The analysis revealed a significant main effect of gender of the participants indicating that male participants performed better than female ones (*glmer* estimated accuracy for male vs. female = .98 ± .01 vs. .95 ± .02; *F* (1, 3418) = 5.10, *p* = .02). A significant main effect of orientation was found indicating that upright pictures were better recognized than inverted ones (*glmer* estimated accuracy for upright vs. inverted = .98 ± .01 vs. .96 ± .02; *F* (1, 3418) = 4.30, *p* = .04).

**Fig 3:**
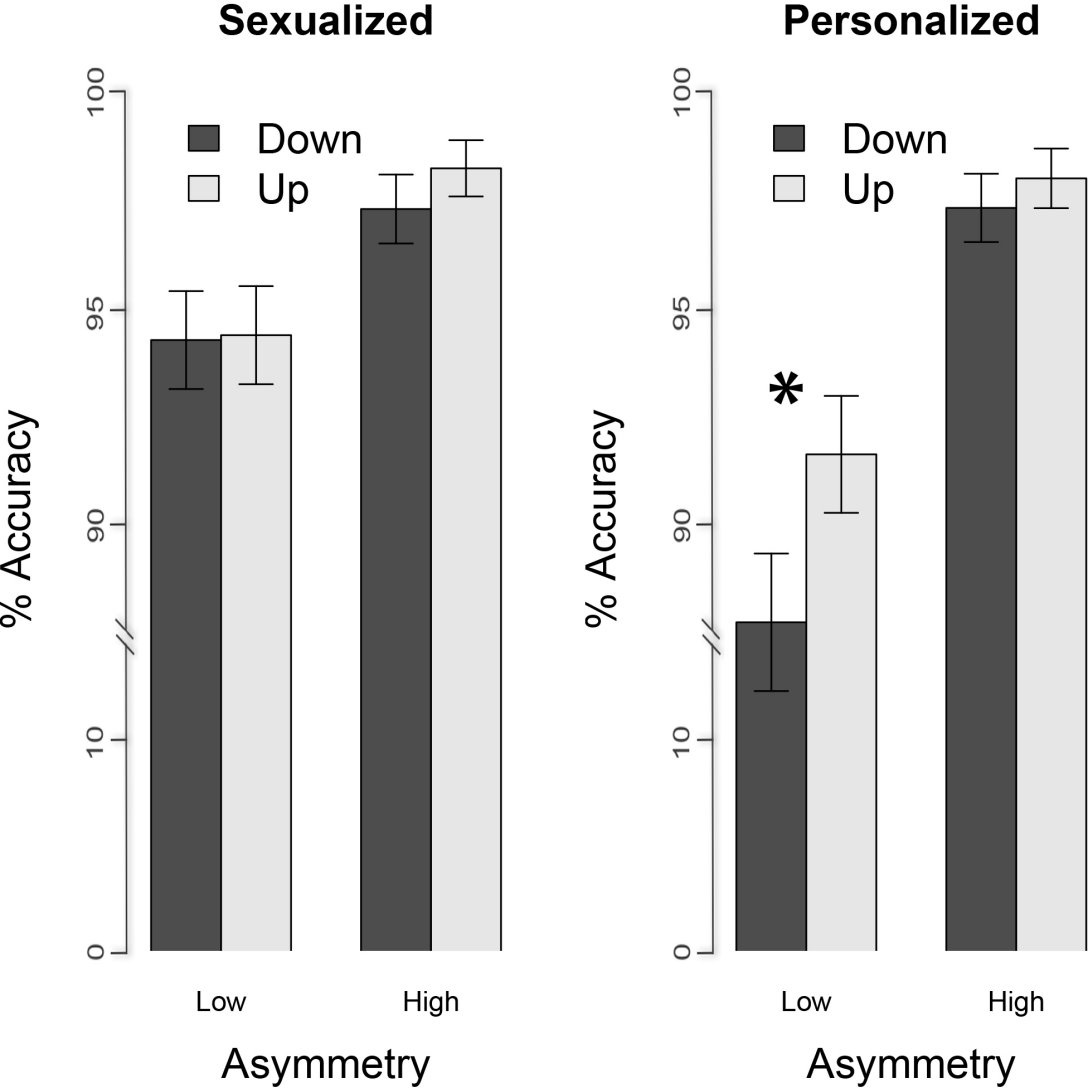
Accuracy. Mean and SE values of the proportion of accuracy as a function of the asymmetry (low, high) and the condition (sexualized, personalized). As by the legend, the color encodes the orientation (up and down). The asterisk indicates the presence of the inversion effect.

A significant main effect of asymmetry was found, *F* (1, 3418) = 57.98, *p* < .001, which was further qualified by the condition, *F* (1, 3418) = 3.54, *p* = .03 (1 tailed). In order to better understand this interaction we looked at the effect of orientation on the accuracy scores separately for each condition and asymmetry of the pictures and run a *glmer* model containing only the orientation as the main effect and the participants as random effect, separately for each condition of each asymmetry subset. Analyses revealed that only in the personalized condition, for the low asymmetrical pictures, pictures were better recognized in the upright orientation than in the inverted one (*glmer* estimated accuracy for upright vs. inverted = .94 ± .03 vs. .90 ± .04; *F* (1, 851) = 4.32, *p* = .04), whereas sexualized pictures were recognized equally well in the upright and in the inverted orientation (*glmer* estimated accuracy for upright vs. inverted = .97 ± .02 vs. .97 ± .02; *F* (1, 846) = 0.01, *p* = .91). As for the high asymmetrical pictures, pictures were recognized equally well in the upright and in the inverted orientation in the personalized condition (*glmer* estimated accuracy for upright vs. inverted = .99 ± .02 vs. .98 ± .02; *F* (1, 861) = 0.37, *p* = .54), as well as in the sexualized condition (*glmer* estimated accuracy for upright vs. inverted = .99 ± .0001 vs. .99 ± .0002; *F* (1, 857) = 0.82, *p* = .37). All the other effects and interactions did not approach the significance level, all *p* > .33 (See Fig 3).

## Experiment 4

In order to test the generalizability of the findings of Experiment 1 and 2 to both gender, we run Experiment 4 with a new dataset of stimuli extended to include also sexualized and personalized male pictures. Moreover, the stimuli were matched for asymmetry to *empirically* rule out the possibility that the findings of Experiments 1 and 2 were due to the different level of asymmetry between conditions (given that a significant inversion effect was observed in both Experiments 1 and 2 for the less asymmetrical pictures: mannequin and personalized conditions). In addition, through an eye-tracker, eye movements were recorded together with the usual behavioral data. Eye-movements (i.e., mean fixation duration (MF) and total number of fixations (NF)), were analyzed to test whether differences in recognition for different categories can be explained by different attention allocation and by different visual sampling of the body parts for the different conditions.

## Method

### Participants

Sixty healthy students (*N* = 30 men and *N* = 30 women; age *M* = 27.52, *SD* = 7.46 years) took part in the present study in exchange for monetary reward. The study was conducted at the University of Vienna. Verbal instructions based on the Italian version were translated to/into German. All participants gave written informed consent before participating in the study, which was approved by the SISSA ethical committee and were treated in accordance with the Declaration of Helsinki. Participants were naïve to the aim of the study and did not participate in similar studies before.

### Stimuli

Participants were engaged in the same task described in Experiment 2. The stimuli dataset consisted of 24 pictures of women and 24 pictures of men wearing a swimsuit or underwear (sexualized condition), 24 pictures of women and 24 pictures of men wearing casual/classic clothes (personalized condition).

**Fig 4:**
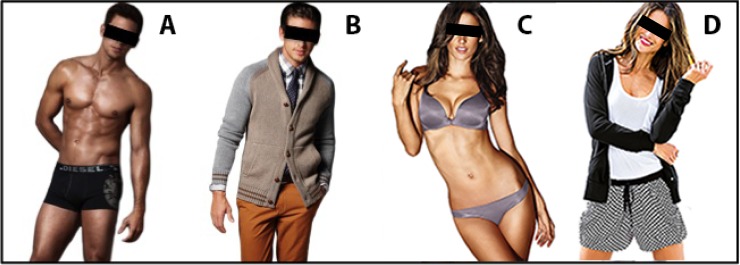
Exemplar target stimuli. Picture of a sexualized man (A), personalized man (B), sexualized woman (C), and personalized woman (D). All stimuli were presented without covering black bars.

Stimuli were collected from free sources from the web. In particular, pictures of the same model portrayed in the sexualized as well as in the personalized condition were selected, in order to avoid possible confounds related to the identity of the model. Both men and women were shown from head to knee, in a standing position, with eyes/face focused on the camera. All pictures were modified to have a white background, the same luminance and the dimension of 397 x 576 Pixels (see Fig 4 for an example of each stimulus´ condition).

In order to test whether the selected pictures were perceived differently in terms of attractiveness, sexiness, intelligence and familiarity, a pretest was conducted with an independent pool of participants. Precisely, 16 naïve participants recruited from the same population of the experimental sample rated all the stimuli on a Likert point scale, ranging from 1 (= not at all) to 6 (= completely) on the perceived level of attractiveness, sexiness, intelligence and familiarity.

A main effect of gender of the picture was observed, meaning that women were rated as sexier, more attractive and familiar than men. Also, a main effect of condition was observed, meaning that sexualized pictures were rated as sexier than the personalized ones; while the personalized pictures were rated as more intelligent than the sexualized ones (see S1 File for the full statistic).

#### Analysis of the asymmetries of the stimuli

A 2 condition (sexualized, personalized) x 2 gender of the picture (male, female) ANOVA was carried out on the Average Axes values (mean of the eyes, shoulders, elbows, hands and hips axes). Both main effects and interactions did not approach the significance level *p* > .19, indicating that there were no differences in terms of asymmetry between the gender of the pictures and the two conditions (See S1 File for the full statistic).

### Procedure

Participants’ visual acuity and oculomotor dominance were assessed. Before starting the experiment, their heads were stabilized with a chin and forehead rest to minimize movements. After successful calibration and validation (9-point) participants saw upright and inverted pictures of sexualized and personalized men and women for 250ms and subsequently had to indicate which of two pictures (original, mirrored version) they had just seen. Eye movements were recorded via an EyeLink 1000 Desktop Mount eye tracker (SR Research Ltd., Mississauga, Ontario, Canada), sampling at 1000 Hz. Viewing was binocular but we recorded the movements of only one eye. Stimuli were shown on a 24” monitor (Samsung SyncMaster 2443BW LCD, widescreen; 16:9; resolution: 1920 x 1200 pixel; refresh rate 60Hz). Participants kept their head in a chin rest such that the viewing distance was 64 cm. The image was centered on the screen and had a size of 397 x 576 Pixels.

### Eye movement data analysis

We defined areas of interest (AOI) per stimulus covering the head, the breast and the pubic region of the depicted persons to analyze whether fixating one of these regions was essential for the decision. For the breast and pubic region, the AOIs were rectangular and both had the same size within one picture. Between pictures the sizes ranged from 130 to 180 pixels in width and 90 to 110 pixels in height. For the face region, the AOIs were circles, the sizes ranged from 110 to 130 pixels’ diameter.

Fixation reports provided by the eye tracker company were filtered using Matlab (version R2012b) to derive fixations. The analyses were focused on three AOIs of the first presented picture in the task. Fixations within the AOIs were analyzed along the main factors (i.e., gender, condition and orientation) for the following dependent variables: mean fixation duration (MF) and mean number of fixations (NF). Given the exploratory nature of the eye-tracking paradigm (due to the very short presentation time), a two-steps analysis approach was chosen. First, in order to check the validity of our procedure, an analysis of the eye movements (MF and NF) of the upright images was performed. This allowed us to compare our results with the existing literature so far available [37, 38, 46]. Next, given the complexity of the model, and because our main goal was to compare the size of the inversion effect between the different conditions (objectified, personalized), a second analysis was performed on the complete set of images, separately for each gender of the picture (male and female).

## Results

### Accuracy

Individual accuracy in the matching task was analyzed with the same settings described in Experiment 1 and 2, using a *generalized linear mixed effect* model (*glmer* R package) with an independent random intercept for every subject and with condition (sexualized, personalized), orientation (upright, inverted), gender of the picture (male, female) and gender of the participant (male, female) as fixed effects. In order to optimize the statistical validity of our dataset we decided to exclude from the analysis participants collecting a performance below the chance level (i.e., 75% correct) in more than 37.5% of tested experimental conditions (n = 8). This results in the exclusion of 8 out of the 60 participants. After the application of this exclusion criteria, we performed an outlier analysis on individual pattern of correct/ incorrect responses: no trials were removed from the analyses as none of the considered individual binary responses deviated more than 4 SD from the individual best fitting *glmer* model, including all interaction terms as fixed factors (4992 trials in total).

The only statistically meaningful outcome of the analysis regards a main effect of orientation, *F* (1, 4991) = 3.69, *p* = .05, with upright pictures (*glmer* estimated accuracy = .93 ± .01) being, on average, better recognized than inverted ones (*glmer* estimated accuracy = .92 ± .02).

**Fig 5:**
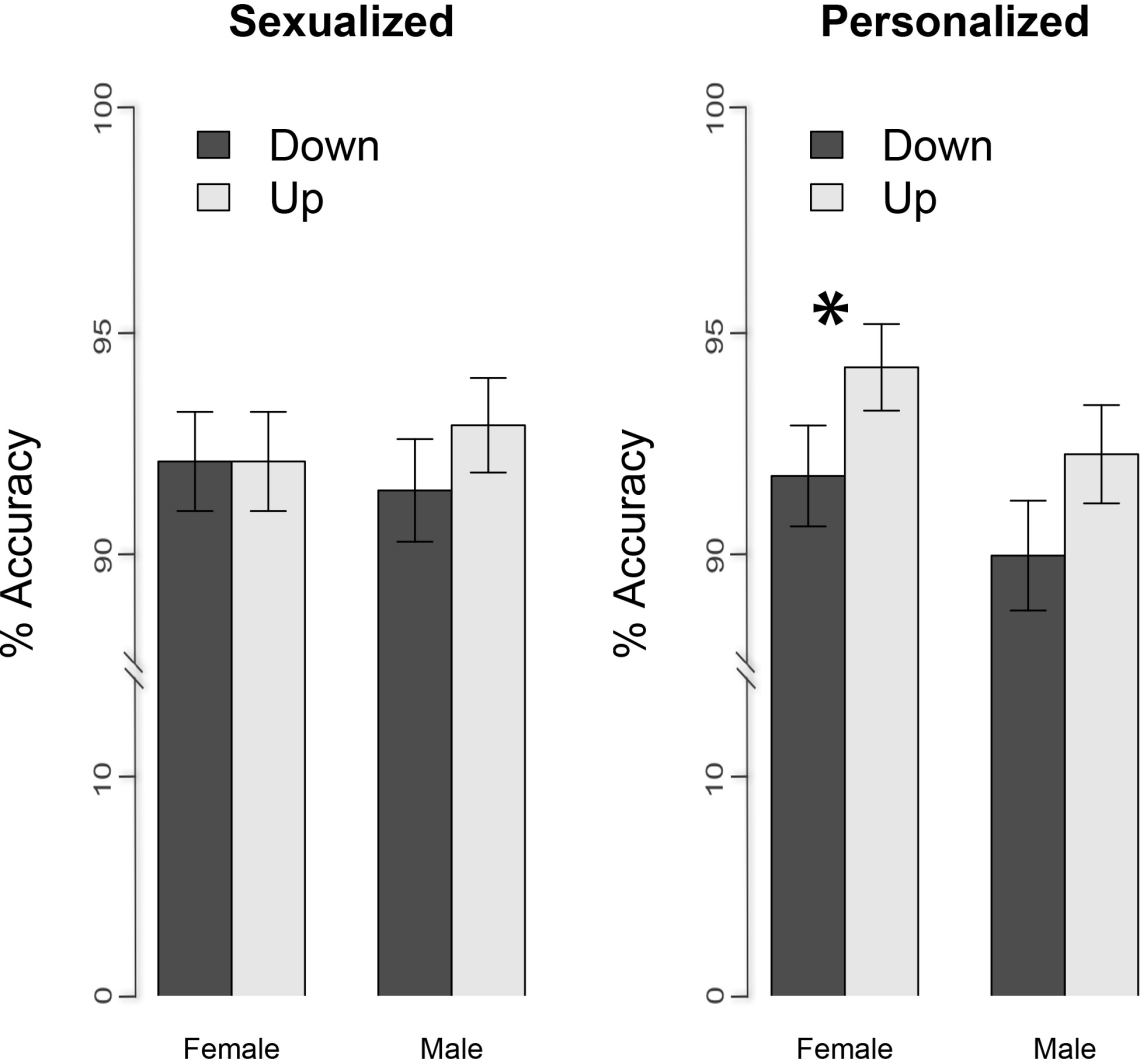
Accuracy. Mean and SE values of the proportion of accuracy as a function of the gender of the picture (male, female), and the condition (sexualized, personalized). As by the legend, the color encodes the orientation (up and down). The asterisk indicates the presence of the inversion effect.

Given the complexity of the model, we run a *glmer* model containing only the orientation as the main effect and the participants as random effect, separately for each condition of each gender of the picture subset. We rely on 1 tailed test as the direction of the expected effect, namely a significant inversion effect for the female personalized images, was already expected on the basis of results of Experiment 1 and 2. Analyses (1 tailed) revealed that in the personalized condition female pictures were better recognized in the upright than the inverted orientation (*glmer* estimated accuracy for upright vs. inverted = .94 ± .02 vs. .92 ± .01; *F* (1, 1247) = 2.76, *p* = .05) while male pictures were recognized equally well in the two orientations (*glmer* estimated accuracy for upright vs. inverted = .93 ± .02 vs. .91 ± .03; *F* (1, 1247) = 1.93, *p* = .08) even if the trend suggests better recognition for the upright as compared to the inverted orientation. As for the sexualized condition, female pictures (*glmer* estimated accuracy for upright vs. inverted = .92 ± .02 vs. .92 ± .02; *F* (1, 1247) = 0.01, *p* = 1) as well as male pictures (*glmer* estimated accuracy for upright vs. inverted = .94 ± .02 vs. .92 ± .03; *F* (1, 1247) = 0.95, *p* = .17) were equally recognized in the two orientations. All the other effects and interactions did not approach the significance level, all *p* > .09 (See Fig 5).

See S1 File for an additional analysis on the moderating role of the asymmetry in this dataset.

### Eye movement data

#### Preliminary analysis

**Fig 6:**
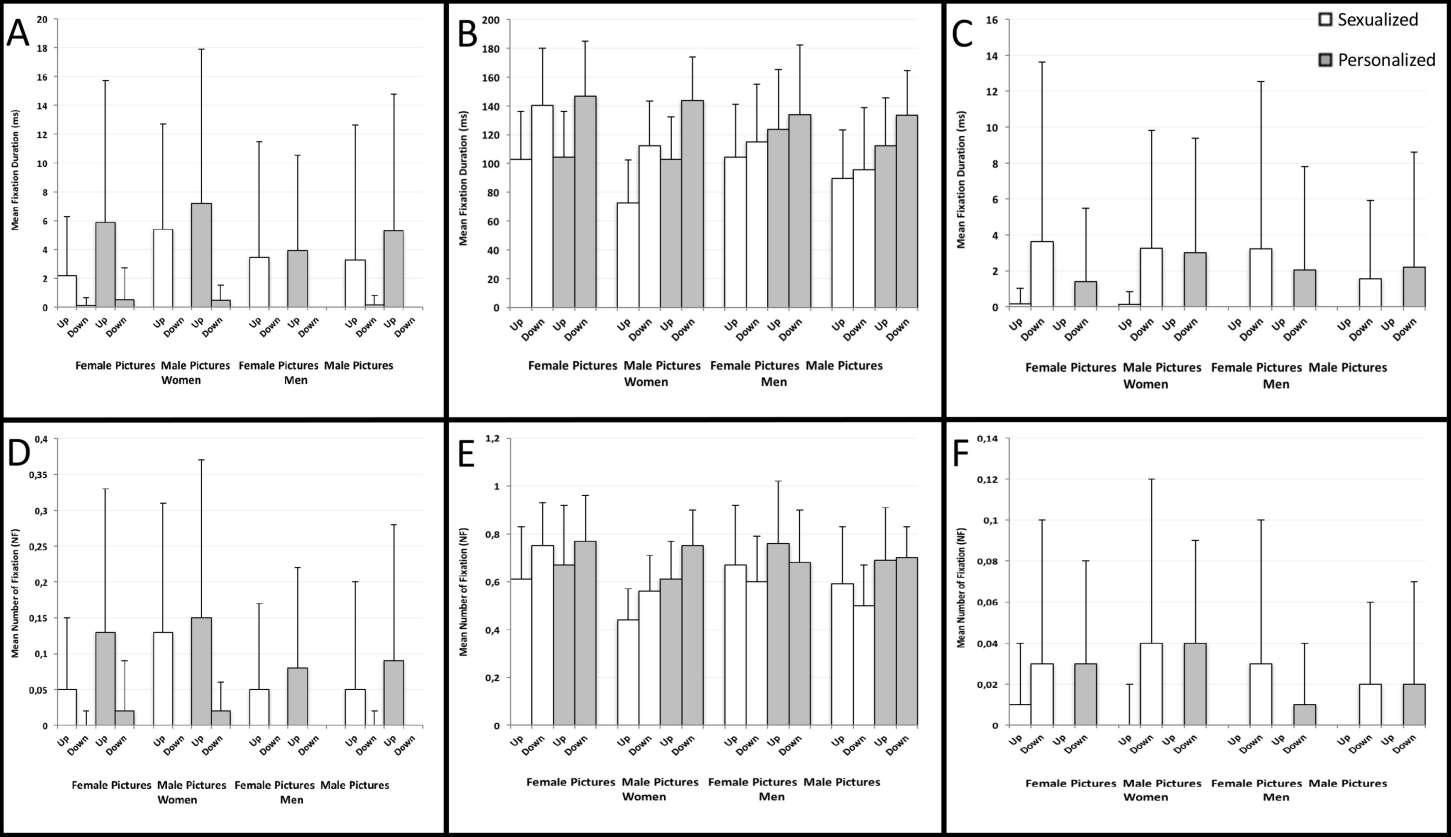
Mean fixation duration and mean number of fixation. Mean and SD values of the Mean fixation duration (Panel A, B, C) and the Number of fixation (Panel D, E, F) split by gender of the participant (female, male), gender of the picture (female, male), condition (sexualized, personalized) and orientation (up and down), are reported separately for each AOI. Panel A &D Face AOI, Panel B &E Breast AOI, Panel C &F Pelvic AOI.

A mixed-model ANOVA with the within-subject factors: condition (sexualized, personalized), gender of the picture (male, female), and the between-subject factor: gender of the participant (male, female) was carried out on the eye movement data (MF and NF) separately for each AOI (see Fig 6 in the main text and Table F and Table G in S1 File).

#### Mean fixation duration (MF)

**Face AOI:** A main effect of gender of the picture was found, F (1, 50) = 7.24, p = .01, *η*_*p*_^*2*^ = .13, meaning that the Face AOI of the male pictures was fixated longer than the female ones. A main effect of condition was found, F (1, 50) = 7.04, *p* = .01, *η*_*p*_^*2*^ = .12, meaning that the Face AOI of the personalized pictures was fixated longer than the sexualized ones. All the other effects and interactions did not approach the significance level (*p* > .08).

**Breast AOI:** A main effect of gender of the picture was found, *F* (1, 50) = 15.12, *p* < .001, *η*_*p*_^*2*^ = .23, meaning that the Breast AOI of female pictures was fixated longer than the male ones. A main effect of condition was found, *F* (1, 50) = 30.33, *p* < .001, *η*_*p*_^*2*^ = .38, meaning that the Breast AOI of the personalized pictures was fixated longer than the sexualized ones.

An interaction of condition x gender of the picture was found, *F* (1, 50) = 5.56, *p* = .02, *η*_*p*_^*2*^ = .10, meaning that only in the sexualized condition the Breast AOI of the female pictures was fixated longer than the male pictures (*p* < .001) while in the personalized condition the Breast AOI of the female pictures was fixated equally longer than the male pictures (*p* = .22). All the other effects and interactions did not approach the significance level (*p* > .07).

**Pelvic AOI:** All the effects and interactions did not approach the significance level *p* > .32.

#### Mean number of fixations (NF)

**Face AOI:** A main effect of gender of the picture was found, F (1, 50) = 6.75, p = .01, *η*_*p*_^*2*^ = .12, meaning that the Face AOI of the male pictures was fixated more often than the female ones. A main effect of condition was found, F (1, 50) = 12.32, p = .001, *η*_*p*_^*2*^ = .20, meaning that the Face AOI of the personalized pictures was fixated more often than the sexualized ones. An interaction of gender of the participant x gender of the picture was found, F (1, 50) = 3.95, p = .05, *η*_*p*_^*2*^ = .07, meaning that only female participants fixated the Face AOI of the male pictures more often than the female ones (*p* = .002). All the other effects and interactions did not approach the significance level (*p* > .12).

**Breast AOI:** A main effect of gender of the picture was found, *F* (1, 50) = 15.16, *p* < .001, *η*_*p*_^*2*^ = .23, meaning that the Breast AOI of female pictures was fixated more times than the male ones. A main effect of condition was found, *F* (1, 50) = 21.63, *p* < .001, *x*_*p*_^*2*^ = .30, meaning that the Breast AOI of the personalized pictures was fixated more times than the sexualized ones. A main effect of the gender of the participants was found, *F* (1, 50) = 3.89, *p* = .05, *η*_*p*_^*2*^ = .07, meaning that male participants fixated the Breast AOI more times than the female participants. All the other effects and interactions did not approach the significance level (*p* > .10).

**Pelvic AOI:** All the effects and interactions did not approach the significance level *p* > .32.

#### Analysis of the inversion effect

In order to reduce the complexity of the model, a mixed-model ANOVA with the within subject factors: condition (sexualized, personalized), orientation (upright, inverted), and between subject factor gender of the participant (male, female) was carried out on the eye movement data (MF and NF) separately for each AOI and for each gender of the picture (but see S1 File for the complete model) (see Fig 6 in the main text and Table F and Table G in S1 File).

#### Female pictures: Mean fixation duration (MF)

**Face AOI:** A main effect of condition was found, F (1, 50) = 4.79, *p* = .03, *η*_*p*_^*2*^ = .09, meaning that the Face AOI of the personalized pictures was fixated longer than the sexualized ones. A main effect of orientation was found, F (1, 50) = 18.49, p < .001, *η*_*p*_^*2*^ = .27, meaning that the Face AOI of the upright pictures was fixated longer than the inverted ones. An interaction of condition x orientation was also found, *F* (1, 50) = 4.20, *p* = .05, *η*_*p*_^*2*^ = .08, meaning that the personalized pictures were fixated longer in the Face AOI than the sexualized pictures only when presented in the upright orientation (*p* = .04) as compared to the inverted one (*p* = .22). All the other effects and interactions did not approach the significance level (*p* > .09).

**Breast AOI:** A main effect of condition was found, *F* (1, 50) = 10.36, *p* = .002, *η*_*p*_^*2*^ = .17, meaning that the Breast AOI of the personalized pictures was fixated longer than the sexualized ones. A main effect of the orientation was found *F* (1, 50) = 10.14, *p* = .002, *η*_*p*_^*2*^ = .17 meaning that the Breast AOI of the inverted pictures was fixated longer than the upright ones. An interaction of condition x gender of the participant was found, *F* (1, 50) = 4.49, *p* = .04, *η*_*p*_^*2*^ = .08, meaning that only female participants fixated the Breast AOI of the inverted pictures longer than the upright ones (*p* < .001) while the male participants fixated the Breast AOI equally longer in the two orientations (*p* = .35). All the other effects and interactions did not approach the significance level (*p* > .07).

**Pelvic AOI:** A main effect of orientation was found *F* (1, 50) = 8.38, *p* = .01, *η*_*p*_^*2*^ = .14, meaning that the Pelvic AOI of the inverted pictures was fixated longer than the upright ones. All the other effects and interactions did not approach the significance level *p* > .16.

#### Female pictures: Mean number of fixations (NF)

**Face AOI:** A main effect of condition was found, F (1, 50) = 7.89, p = .01, *η*_*p*_^*2*^ = .14, meaning that the Face AOI of the personalized pictures was fixated more times than the sexualized ones. A main effect of orientation was found, F (1, 50) = 19.98, p < .001, *η*_*p*_^*2*^ = .29, meaning that the Face AOI of the upright pictures was fixated more times than the inverted ones.

An interaction of condition x orientation was also found, *F* (1, 50) = 7.76, *p* = .01, *η*_*p*_^*2*^ = .13, meaning that the personalized pictures were fixated more times in the Face AOI than the sexualized pictures only when presented in the upright orientation (*p* = .01) as compared to the inverted one (*p* = .21). All the other effects and interactions did not approach the significance level (*p* > .09).

**Breast AOI:** A main effect of condition was found, *F* (1, 50) = 8.97, *p* = .004, *η*_*p*_^*2*^ = .15, meaning that the Breast AOI of the personalized pictures was fixated more often than the sexualized ones. An interaction of orientation x gender of the participant was found, *F* (1, 50) = 4.26, *p* = .04, *η*_*p*_^*2*^ = .08, meaning that female participants fixated the Breast AOI of the female pictures in the inverted orientation more time than the male participants (*p* = .02), whereas pictures in the upright orientation were equally fixated by male and female participants (*p* = .23). All the other effects and interactions did not approach the significance level (*p* > .22).

**Pelvic AOI:** A main effect of orientation was found, *F* (1, 50) = 11.67, *p* = .001, *η*_*p*_^*2*^ = .19, meaning that the Pelvic AOI of the inverted pictures was fixated more times than in the upright ones. All the other effects and interactions did not approach the significance level (*p* > .12).

#### Male pictures: Mean fixation duration (MF)

**Face AOI:** A main effect of condition was found, F (1, 50) = 6.36, *p* = .02, *η*_*p*_^*2*^ = .11, meaning that the Face AOI of the personalized pictures was fixated longer than of the sexualized ones. A main effect of orientation was found, F (1, 50) = 17.61, p < .001, *η*_*p*_^*2*^ = .26, meaning that the Face AOI of the upright pictures was fixated longer than the inverted ones. An interaction of condition x orientation was also found, *F* (1, 50) = 4.55, *p* = .04, *η*_*p*_^*2*^ = .08, meaning that the personalized pictures were fixated longer in the Face AOI than the sexualized pictures only when presented in the upright orientation (*p* = .02) as compared to the inverted one (*p* = .18). All the other effects and interactions did not approach the significance level (*p* > .38).

**Breast AOI:** A main effect of condition was found, *F* (1, 50) = 117.50, *p* < .001, *η*_*p*_^*2*^ = .70, meaning that the Breast AOI of the personalized pictures was fixated longer than the sexualized ones. A main effect of the orientation was found *F* (1, 50) = 20.21, *p* < .001, *η*_*p*_^*2*^ = .29 meaning that the Breast AOI of the inverted pictures was fixated longer than the upright ones. An interaction of orientation x gender of the participant was found, *F* (1, 50) = 4.89, *p* = .03, *η*_*p*_^*2*^ = .09, meaning that only female participants fixated the Breast AOI of the inverted pictures longer than the upright ones (*p* < .001) while the male participants fixated the Breast AOI equally longer in the two orientations (*p* = .11). All the other effects and interactions did not approach the significance level (*p* > .25).

**Pelvic AOI:** A main effect of orientation was found *F* (1, 50) = 14.59, *p* < .001, *η*_*p*_^*2*^ = .23, meaning that the Pelvic AOI of the inverted pictures was fixated longer than the upright ones. All the other effects and interactions did not approach the significance level *p* > .30.

#### Male pictures: Mean number of fixations (NF)

**Face AOI:** A main effect of condition was found, F (1, 50) = 7.86, p = .01, *η*_*p*_^*2*^ = .14, meaning that the Face AOI of the personalized pictures was fixated more times than the sexualized ones. A main effect of orientation was found, F (1, 50) = 16.73, p < .001, *η*_*p*_^*2*^ = .25, meaning that the Face AOI of the upright pictures was fixated more times than the inverted ones. All the other effects and interactions did not approach the significance level (*p* > .09).

**Breast AOI:** A main effect of condition was found, *F* (1, 50) = 103.40, *p* < .001, *η*_*p*_^*2*^ = .67, meaning that the Breast AOI of the personalized pictures was fixated more often than the sexualized ones. An interaction of orientation x gender of the participant was found, *F* (1, 50) = 7.41, *p* = .01, *η*_*p*_^*2*^ = .13, meaning that female participants fixated the Breast AOI of the inverted male pictures more times than the upright ones often (*p* = .01), whereas male participants fixated the Breast AOI of the male pictures in the upright and inverted orientation equally often (*p* = .36). All the other effects and interactions did not approach the significance level (*p* > .13).

**Pelvic AOI:** A main effect of orientation was found, *F* (1, 50) = 16.31, *p* < .001, *η*_*p*_^*2*^ = .25, meaning that the Pelvic AOI of the inverted pictures was fixated more times than the upright ones. All the other effects and interactions did not approach the significance level (*p* > .13).

## Discussion

So far, no clear conclusion could be drawn regarding the nature of the processes involved in the perception of sexualized women according to sexual objectification theory (see [11, 12, 22, 24, 25, 29]. Across four experiments, we were able to show that the degree of sexualization, the visual properties of the stimuli as well as participants' attention biases contribute to the presence/absence of the inversion effect. In particular, we observed a difference in the visual exploration of the stimuli according to the degree of sexualization of the images, as reflected in reduced exploration of the face region only in inverted personalized stimuli compared to the upright ones, with the corresponding presence of inversion effect. More importantly, we were able to show that the inversion effect is moderated (and not mediated) by the visual properties of the stimuli, namely the degree to which the stimuli differ in terms of asymmetry. Only when asymmetry is low, the category of the stimuli (sexualized vs. personalized) influences the processing style of recognition. Finally we observed that the level of sexualization impacts the occurrence of the SBIE, similarly for both genders.

### Conceptual replication of the sexualized-body inversion effect

The first step of our study was to establish the presence of the SBIE (i.e sexualized women do not show an inversion effect, indicating an analytical processing style) and to characterized the impact of the level of sexualization on the SBIE (i.e personalized women do show an inversion effect, indicating a configural processing style) by using a set of stimuli comparable to the one of Bernard and colleagues [11, 12]. This allowed showing that the SBIH is supported when stimuli are not controlled for visual properties, such as asymmetry.

In line with the previous literature [22], in Experiments 1 and 2 we observed that the recognition of personalized women is worse in the inverted compared to the upright orientation (indicating the presence of an inversion effect), whereas sexualized women were recognized to a similar extent in both the upright and inverted orientation, thus suggesting that the level of sexualization has an impact on the occurrence of the SBIE.

In addition to Bernard et al. [11, 12], we showed that the way in which participants process pictures of sexualized women with different orientations is similar to the way participants recognize pictures of objects such as houses.

### The inversion effect as an indicator of sexualized stimuli processing style

As a second step, we tested the assumption that if sexualized targets lack an inversion effect, they should also be processed as objects. To this aim, in Experiments 1 and 2 we also estimated to which extent the analytical processing style is applied to different types of objects.

We observed that participants were less accurate in processing human body-like objects such as mannequins in the inverted than in the upright orientation. These results indicate that the classification of a stimulus as an object is not a sufficient condition for the absence of an inversion effect (see also Tarr [21, 47]). In fact, human body-like objects, such as mannequins, show the facilitation for upright compared to inverted orientation. This suggests that other features, a part from the semantic category (human vs. object), are responsible for the observed effect. One candidate feature, which is possibly independent from nakedness, is the presence/absence of salient sexual attributes. The inversion effect has been taken as an indirect indicator of configural processing in a variety of studies adopting quite heterogeneous stimuli and tasks [10, 14–19]. This phenomenon has been mostly observed for faces (FIE or “Face inversion effect”) [48], but it has also been reported for bodies (BIE or “Body Inversion Effect”). However, while a drop of accuracy recognition of inverted full body stimuli has been consistently found [26, 49–55], results are mixed for headless bodies [51, 54, 56, 57]. A huge uncertainty still exists on the mechanism underling the occurrence of the inversion effect in bodies, and two plausible explanations have been put forward so far. On one hand, the inversion effect could arise from the disruption of the internal representation of the facial features in its prototypical orientation [26, 55] On the other hand, findings from Brandman & Yovel [56], suggest that the BIE can also be triggered for faceless stimuli presented in a body context, suggesting that other mechanisms a part from facial features and their relationship are responsible for the effect. Beside the state of the art, the present work does not provide any additional information on the validity of one of the two hypotheses; therefore these two theories still leave an open debate on which mechanism can account for the inversion effect.

### An alternative interpretation of the SBIE

Following Tarr´s criticisms [21], we considered several other factors that could have alternatively explained the data observed in Experiments 1 and 2 (i.e. *familiarity*, *asymmetry*, *attention allocation*). First, the pretest revealed that personalized women were perceived as more familiar than sexualized women and mannequins, thus putting forward the idea that our findings could have been driven by differences in stimulus-familiarity of the targets. However, since mannequins (which are unfamiliar to participants) showed the same pattern of result as personalized women, this hypothesis should be discarded.

Second, in order to ascertain that findings of Experiments 1 and 2 could be due to the different degree of asymmetry observed between conditions, a mediational analysis was performed for both experiments. Results showed the absence of a mediating role of asymmetry in shaping the SBIE: the difference in recognition of the targets in the two orientations was not driven by the stimulus asymmetry. However mediational analyses are not sufficient to establish a clear role of the asymmetry in shaping the inversion effect.

In order to test the moderating role of stimulus symmetry, this stimulus feature was systematically varied in Experiment 3. This allowed to further assess the boundary conditions of the SBIE. Results revealed that highly asymmetrical stimuli were equally well recognized in the two orientations regardless of the condition. More importantly, the effect of the condition was evident at low level of asymmetry, indicating the presence of the inversion effect for personalized but not for sexualized images. Therefore, visual properties strongly influence stimulus recognition by facilitating visual matching for high asymmetrical stimuli. However, and in contrast to Schmidt and Kistemaker’s [22] conclusion, the present work confirms that the level of sexualization has an impact on the SBIE beyond the asymmetry confound.

Experiment 4 was subsequently performed with a new set of stimuli matched for asymmetry. Results confirmed the findings of Experiment 3 as indicated by the presence of the inversion effect only for pictures in the personalized condition while pictures in the sexualized condition were recognized equally well in the upright and inverted orientation. Importantly, in line with recent neural evidences [29], the results also indicate that it is indeed the sexualized nature and not the gender of the stimulus that triggers the different processing style, given that the effect was found for both sexualized women and men. Note thought that the inversion effect felt short of significant in the personalized male sample, somehow dampening the conclusion of a clear effect of the level of sexualization on the SBIH for the male gender. More studies are needed to replicate these findings.

Why did Schmidt and colleagues [22] fail to observe the SBIE? Since stimulus symmetry in Experiment 4 was manipulated similarly to Schmidt and colleagues, the discrepancy between the two studies may be due to differences in experimental stimuli. While Schmidt and colleagues used pictures of women and men that were fully naked, we instead employed pictures of men and women in underwear or bikini. It is possible that naked stimuli do not trigger the same sexualized perception of the target, with the concomitant analytic processing style and the SBIE, as the stimuli used here. The very specific difference between naked stimuli and bodies wearing underwear still has to be established in future research. A second possible explanation may relate to the postures adopted by the different stimuli used, independently from asymmetry. Different postures can convey specific meanings that affect the perception of the body, leading to the sexualization of the target. Future studies should systematically address this point in order to clarify the role of body posture in the emergence of the SBIE.

#### The role of attention biases on the occurrence of the SBIE

Finally, the use of an eye-tracker in Experiment 4 allowed us to measure how pictures were inspected in all the conditions, and to assess the role of attention biases in the occurrence of the SBIE. Even if fast saccades have been previously reported [40], to our knowledge this is the first study using short exposure times in combination with an eye tracking device. Therefore, in order to check the validity of our data, a first analysis was performed on the upward images, in order to allow for the comparison with the previous literature. In line with the work by Nummenmaa [37], we observed that people tend to focus (in terms of both duration and number of fixations) more on the face region of the personalized pictures in comparison to sexualized pictures, suggesting that the sexualized representation of a target shifts the focus of attention away from the face. Moreover, the male facial region was fixated more times than the female one indicating that the shift of attention towards the face is a phenomenon that is more present when exploring male targets. In line with this, we also observed a stronger focus on the breast region for female compared to male images. Interestingly, our data indicate longer duration and higher number of fixations on the breast of personalized compared to objectified targets. The result, even if it seems in contradiction to Nummenmaa´s [37] main finding, is possibly due to the different paradigm we used and specifically to the fact that the location of the breast AOI in our task was in proximity of the fixation cross preceding the images (see Figure D in S1 File). This, in combination with the very short time window (250ms), may have led to the tendency to gaze longer at the center of the personalized image, before moving the eyes. Note though that the personalized condition was still characterized by a higher number and a longer duration of fixations to the Face AOI, indicating that the two AOIs did not cancel each other out. Finally, the absence of saccades to the pelvic region indicates that with this short stimulus presentation time participants mostly shifted their focus of attention from the center (breast) to the upper zone (face) of the images.

As a second step, we analyzed the eye-tracker data separately for female and male images both for inverted and upward images. This allowed us to explore the effect of sexualization on the inversion effect.

An effect of orientation was found, in that compared to the inverted pictures upright female and male pictures were fixated longer and more frequently in the face region. Inverted images were longer and more frequently fixated in the breast and pelvic region. In line with the previous analysis, these findings reflect participants’ tendency to move the eyes from the center (breast) to the upper part of the pictures (the face for the upright and the pelvis for the inverted images). A main effect of condition was found for both, male and female pictures, indicating that the face and breast of the personalized targets were fixated longer and more frequently (for the faces only) than those of the sexualized ones. A crucial *condition by orientation* effect was found, indicating that pictures of both sexes were fixated longer in the face AOI in the personalized compared to the sexualized condition but only when presented upright. This suggests that participants’ tendency to explore the face of personalized stimuli was suppressed when stimuli were shown upside down. Taken together, the data suggest that differences in visual exploration of the stimuli indicate a mechanism responsible for the difference in the occurrence of the inversion effect. Namely, the shift of attention from the face to other parts of the body possibly disrupts the configural processing otherwise applied to the perception of the personalized targets.

### Limitation of the present study

Despite the attempt to control the present set of stimuli for visual properties such as asymmetry, we are aware that additional perceptual factors, which were not controlled in this study, could contribute to the occurrence of the SBIE. For example, as highlighted by Tarr [21], differences between conditions in postures, in the colors and types of clothes could influence the occurrence and the magnitude of the SBIE. The images were chosen to strongly resemble those magazines´ portraits and people that participants of our study would typically encounter in everyday life (see also Civile et al. [24] for a similar concern, p. 209). This choice was made in order to have a high level of ecological validity of the stimuli used. However, this was done at the expenses of possible uncontrolled perceptual differences between the different conditions. Thus, despite the accurate investigation of the asymmetries of the images adopted in every dataset, the presence of those perceptual confounds (i.e., complexity, colors etc.) should be considered and controlled for future research.

Another limitation of the present study lays in the adopted operationalization of the concept “sexualized target.” In fact it is possible that, apart from the amount of visible skin, other features can be responsible for the sexualization of the target, such as the clothing style, poses etc. Future studies are needed to clarify the conditions under which the phenomenon occurs or not.

Finally, we would like to highlight that the brief stimulus presentation, necessary to detect the SBIE, has never been used in combination with eye-tracking. Even if the results are in line with our hypothesis and with previous findings, future studies should explore if this pattern generally persist with longer exploration times.

### Conclusion

In a series of four experiments, we were able to show that the presence or absence of the inversion effect (index of a configural/analytical processing style), previously associated with the processing of sexualized women, is strongly influenced by several factors, such as the level of sexualization of the target, the symmetry of the stimuli, and the visual exploration strategy adopted by the participants. In particular, we observed that the analytical processing style is not linked to the semantic classification “object vs. person” but rather applied to the perception of sexualized targets. This suggests that referring to “sexualized” women (or men) is a more precise expression than “objectified” women or men. We also observed that the level of asymmetry of the pictures accounts for some of the variability in the occurrence of the inversion effect, but does not undermine the SBIH. Finally, sexualized pictures are visually explored with a different strategy as indicated by lower number of fixations in the face region compared to the personalized pictures. These differences in the visual exploration of the stimulus may therefore translate in the SBIE by triggering a different processing style (analytical *vs*. configural).

In conclusion, the findings of the present study contribute to the ongoing debate by tipping the balance in favour of the SBIH. The SBIE indeed cannot be simply reduced to a methodological artefact, given that the sexualization of the target is able to trigger an analytical processing style, on top of a moderating effect of by the stimulus asymmetry. Our results also suggest that future studies should address other possible conditions under which this phenomenon appears, by considering both the visual properties of the stimuli (i.e. clothing, postures) and the individual differences of the perceiver.

## Supporting information

S1 File (DOCX)

S2 File Experiment 1 data. (TXT)

S3 File Experiment 2 data. (TXT)

S4 File Experiment 3 data. (TXT)

S5 File Experiment 4 data. (TXT)
