# Supplementary material for: Understanding the mechanisms behind the sexualized-body inversion hypothesis: The role of asymmetry and attention biases
Source: PLoS One. 2018 Apr 5;13(4):e0193944. doi: 10.1371/journal.pone.0193944 (PMC5886406; doi:10.1371/journal.pone.0193944)
Supplement: S1 File — (DOCX) [file pone.0193944.s001.docx]

**Supplementary Materials**

**Supplementary figures**

**Fig A. Exemplar stimulus for the House control condition.**


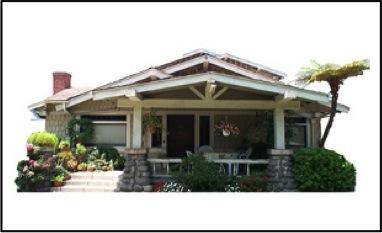


**Fig B. Exemplar trial for the visual recognition task.** For each trial, a picture appeared in the middle of the computer screen for 250 ms, followed by a blank screen for 1000 ms. Immediately after, participants were presented with two pictures, one on the left side of the screen and one on the right, in which one of the two was the original picture, and the other was its left-right mirrored version. Participants were requested to indicate which one of the two pictures was the one they had previously seen. All stimuli were presented without the distortion bars on the face.

**
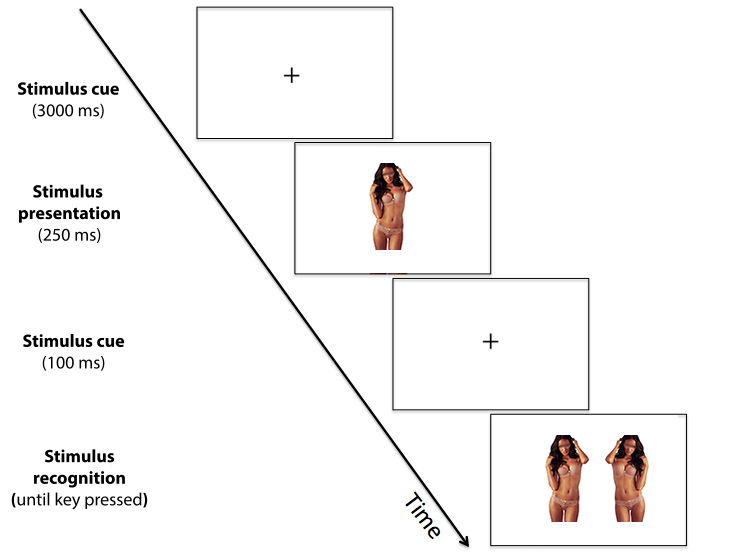
**

**Fig C. Accuracy scores for Experiment 1.** Mean and SE values of the accuracy score of houses and target stimuli split by group (sexualized, personalized and mannequin) and orientation (up and down) are reported. The asterisk indicates the presence of the inversion effect.

**
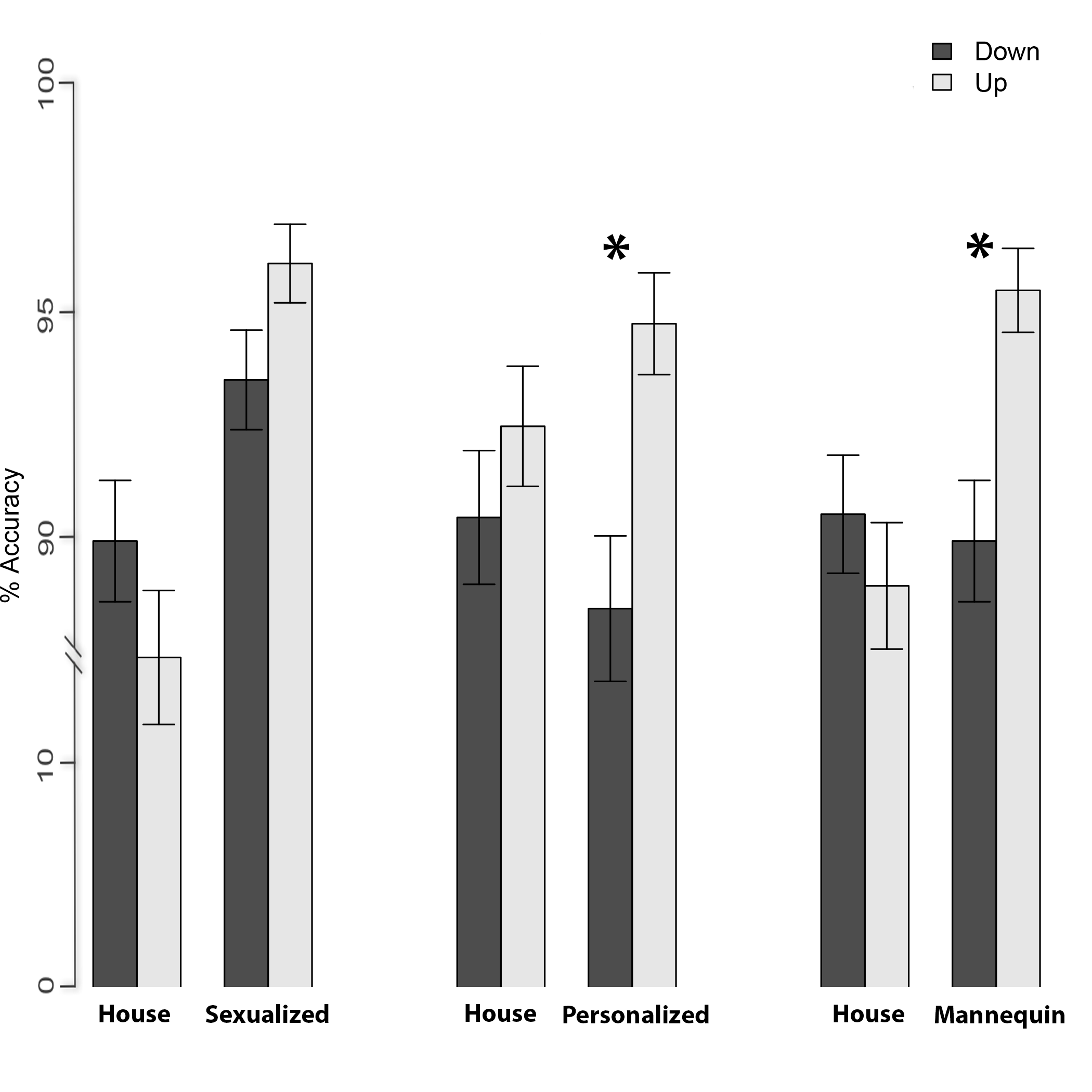
**

**Fig D. Fixation cross location on the screen immediately preceding the target images.** The red dot indicated where the fixation cross appeared immediately before the target presentation.

**
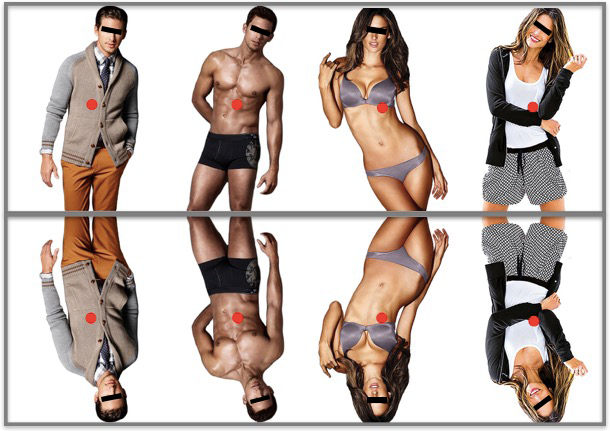
**

**Supplementary Tables**

**Table A. Picture ratings for Experiment 1**

| Dimensions | Gender of Participants | Sexualized | Personalized | Mannequin |
| --- | --- | --- | --- | --- |
| Sexiness | Women | 31.39 (0.51) | 30.31 (0.46) | 28.54 (0.23) |
|  | Men | 31.86 (0.48) | 30.59 (0.38) | 28.43 (0.18) |
| Attractiveness | Women | 31.11 (0.52) | 30.96 (0.46) | 28.65 (0.16) |
|  | Men | 31.51 (0.39) | 31.21 (0.48) | 28.49 (0.22) |
| Intelligence | Women | 30.00 (0.54) | 30.86 (0.37) | 28.64 (0.16) |
|  | Men | 30.15 (0.39) | 30.94 (0.35) | 28.49 (0.12) |
| Familiarity | Women | 29.72 (0.35) | 30.45 (0.38) | 29.85 (0.20) |
|  | Men | 30.77 (0.35) | 31.01 (0.36) | 30.40 (0.23) |

Note. Mean values and standard errors (in brackets) for the ratings for each dimension split by group and gender of the participant.

**Table B. Picture ratings for Experiment 4**

| Picture Gender | Dimension | Participant Gender | Sexualized | Personalized |
| --- | --- | --- | --- | --- |
| Female | Sexiness | Women | 4.30 (0.53) | 3.62 (0.6) |
|  |  | Men | 4.13 (0.41) | 3.38 (0.59) |
|  | Attractiveness | Women | 4.49 (0.5) | 4.23 (0.56) |
|  |  | Men | 4.1 (0.44) | 4.08 (0.59) |
|  | Intelligence | Women | 3.68 (0.27) | 4.06 (0.36) |
|  |  | Men | 2.96 (0.34) | 3.53 (0.34) |
|  | Familiarity | Women | 1.89 (0.65) | 1.9 (0.76) |
|  |  | Men | 1.92 (0.43) | 1.81 (0.37) |
| Male | Sexiness | Women | 3.38 (0.55) | 3.37 (0.65) |
|  |  | Men | 3.26 (0.67) | 3.05 (0.73) |
|  | Attractiveness | Women | 3.7 (0.53) | 3.8 (0.63) |
|  |  | Men | 3.23 (0.66) | 3.48 (0.84) |
|  | Intelligence | Women | 3.48 (0.35) | 3.87 (0.46) |
|  |  | Men | 2.94 (0.99) | 3.36 (0.55) |
|  | Familiarity | Women | 1.52 (0.19) | 1.53 (0.19) |
|  |  | Men | 1.68 (0.35) | 1.49 (0.33) |

Note. Mean and standard errors (in brackets) for the ratings for each dimension split by condition, gender of the picture and gender of the participant.

**Table C. Asymmetry values for Experiment 1-2**

| Axes | Mannequin | Sexualized | Personalized |
| --- | --- | --- | --- |
| Shoulders | 6.14 (3.58) | 10.41 (7.12) | 11.04 (6.66) |
| Hands | 14.51 (11.53) | 44.03 (25.82) | 27.72 (27.35) |
| Elbows | 6.73 (4.46) | 19.86 (20.50) | 12.38 (9.53) |
| Hips | 8.48 (5.33) | 15.492 (7.19) | 8.51 (4.52) |
| Ass_Index | 8.97 (4.48) | 22.49 (10.03) | 14.91 (9.32) |

Note. Mean and standard errors (in brackets) for each axis split by condition.

**Table D. Asymmetry values for Experiment 3**

| Axes | Assymetry | Sexualized | Personalized |
| --- | --- | --- | --- |
| Eyes | Low | 3.31 (2.58) | 4.16 (4.13) |
|  | High | 8.60 (8.88) | 16.78 (14.77) |
| Shoulders | Low | 6.73 (2.57) | 4.88 (4.01) |
|  | High | 11.04 (8.31) | 11.90 (5.82) |
| Elbows | Low | 3.36 (3.6) | 4.17 (2.89) |
|  | High | 33.33 (21.49) | 22.78 (15.74) |
| Hands | Low | 5.68 (4.25) | 5.86 (5.49) |
|  | High | 59.58 (14.21) | 60.52 (17.43) |
| Hips | Low | 10.43 (3.37) | 7.26 (4.79) |
|  | High | 17.24 (7.55) | 12.43 (5.26) |
| Ass_Index | Low | 5.9 (1.72) | 5.26 (2.24) |
|  | High | 25.96 (3.87) | 24.88 (3.68) |

Note. Mean and standard errors (in brackets) for each axis split by condition and level of asymmetry.

**Table E. Asymmetry values for Experiment 4**

| Axes | Picture Gender | Sexualized | Personalized |
| --- | --- | --- | --- |
| Shoulders | Women | 9.05 (5.52) | 10.71 (6.34) |
|  | Men | 10.51 (5.66) | 8.63 (8.77) |
| Eyes | Women | 7.7 (11.14) | 9.87 (11.16) |
|  | Men | 9.65 (8.75) | 5.28 (4.06) |
| Hands | Women | 22.29 (24.6) | 26.44 (25.24) |
|  | Men | 22.38 (23.06) | 18.39 (23.74) |
| Elbows | Women | 7.14 (8.7) | 11.61(9.36) |
|  | Men | 11.83 (14.02) | 11.3 (16.82) |
| Hips | Women | 11.72 (4.8) | 9.7 (4.5) |
|  | Men | 5.44 (3.33) | 4.46 (2.84) |
| Ass_Index | Women | 11.58 (6.85) | 13.66 (7.84) |
|  | Men | 11.97 (7.71) | 9.61 (9.88) |

Note. Mean and standard errors (in brackets) for each axis split by condition and gender of the picture.

**Table F. Fixation Duration.**

| **Participant** | | **Picture** | **Condition** | **Orientation** | **Pelvic** | | **Face** | | **Breast** | |
| --- | --- | --- | --- | --- | --- | --- | --- | --- | --- | --- |
| **Female** | **Female** | | **Sex** | **Up** | 0.17 | (0.85) | 2.16 | (4.12) | 102.79 | (33.32) |
|  |  | |  | **Down** | 3.62 | (10.00) | 0.11 | (0.54) | 140.22 | (39.99) |
|  |  | | **Pers** | **Up** | 0.00 | (0.00) | 5.87 | (9.84) | 104.40 | (31.73) |
|  |  | |  | **Down** | 1.40 | (4.08) | 0.51 | (2.20) | 146.54 | (38.22) |
|  | **male** | | **Sex** | **Up** | 0.14 | (0.69) | 5.38 | (7.34) | 72.52 | (29.95) |
|  |  | |  | **Down** | 3.26 | (6.57) | 0.00 | (0.00) | 112.27 | (30.89) |
|  |  | | **Pers** | **Up** | 0.00 | (0.00) | 7.18 | (10.70) | 102.80 | (29.60) |
|  |  | |  | **Down** | 3.00 | (6.39) | 0.47 | (1.06) | 143.56 | (30.27) |
| **Male** | **Female** | | **Sex** | **Up** | 0.00 | (0.00) | 3.46 | (7.99) | 104.18 | (36.75) |
|  |  | |  | **Down** | 3.22 | (9.32) | 0.00 | (0.00) | 114.92 | (40.28) |
|  |  | | **Pers** | **Up** | 0.00 | (0.00) | 3.93 | (6.60) | 123.59 | (41.72) |
|  |  | |  | **Down** | 2.04 | (5.77) | 0.00 | (0.00) | 133.98 | (48.31) |
|  | **Male** | | **Sex** | **Up** | 0.00 | (0.00) | 3.26 | (9.38) | 89.46 | (33.96) |
|  |  | |  | **Down** | 1.54 | (4.39) | 0.13 | (0.67) | 95.56 | (43.07) |
|  |  | | **Pers** | **Up** | 0.00 | (0.00) | 5.29 | (9.49) | 112.31 | (33.44) |
|  |  | |  | **Down** | 2.21 | (6.41) | 0.00 | (0.00) | 133.62 | (30.95) |
| **Total** | **Female** | | **Sex** | **Up** | 0.08 | (0.60) | 2.81 | (6.33) | 103.49 | (34.74) |
|  |  | |  | **Down** | 3.42 | (9.57) | 0.05 | (0.38) | 127.57 | (41.74) |
|  |  | | **Pers** | **Up** | 0.00 | (0.00) | 4.90 | (8.35) | 113.99 | (37.96) |
|  |  | |  | **Down** | 1.72 | (4.96) | 0.26 | (1.56) | 140.26 | (43.59) |
|  | **Male** | | **Sex** | **Up** | 0.07 | (0.49) | 4.32 | (8.41) | 80.99 | (32.84) |
|  |  | |  | **Down** | 2.40 | (5.60) | 0.07 | (0.47) | 103.92 | (38.06) |
|  |  | | **Pers** | **Up** | 0.00 | (0.00) | 6.24 | (10.06) | 107.55 | (31.63) |
|  |  | |  | **Down** | 2.60 | (6.35) | 0.23 | (0.78) | 138.59 | (30.73) |

Note. Mean and standard deviation (in brackets) for the fixation duration split by AOIs, condition (sexualized personalized), orientation (up, down), gender of the picture (male, female) and gender of the participant (male, female).

**Table G. Number of fixation.**

| **Participant** | | **Picture** | **Condition** | **Orientation** | **Pelvic** | | **Face** | | **Breast** | |
| --- | --- | --- | --- | --- | --- | --- | --- | --- | --- | --- |
| **Female** | **Female** | | **Sex** | **Up** | 0.01 | (0.03) | 0.05 | (0.10) | 0.61 | (0.22) |
|  |  | |  | **Down** | 0.03 | (0.07) | 0.00 | (0.02) | 0.75 | (0.18) |
|  |  | | **Pers** | **Up** | 0.00 | (0.00) | 0.13 | (0.20) | 0.67 | (0.25) |
|  |  | |  | **Down** | 0.03 | (0.05) | 0.02 | (0.07) | 0.77 | (0.19) |
|  | **Male** | | **Sex** | **Up** | 0.00 | (0.02) | 0.13 | (0.18) | 0.44 | (0.13) |
|  |  | |  | **Down** | 0.04 | (0.08) | 0.00 | (0.00) | 0.56 | (0.15) |
|  |  | | **Pers** | **Up** | 0.00 | (0.00) | 0.15 | (0.22) | 0.61 | (0.16) |
|  |  | |  | **Down** | 0.04 | (0.05) | 0.02 | (0.04) | 0.75 | (0.15) |
| **Male** | **Female** | | **Sex** | **Up** | 0.00 | (0.00) | 0.05 | (0.12) | 0.67 | (0.25) |
|  |  | |  | **Down** | 0.03 | (0.07) | 0.00 | (0.00) | 0.60 | (0.19) |
|  |  | | **Pers** | **Up** | 0.00 | (0.00) | 0.08 | (0.14) | 0.76 | (0.26) |
|  |  | |  | **Down** | 0.01 | (0.03) | 0.00 | (0.00) | 0.68 | (0.22) |
|  | **Male** | | **Sex** | **Up** | 0.00 | (0.00) | 0.05 | (0.15) | 0.59 | (0.24) |
|  |  | |  | **Down** | 0.02 | (0.04) | 0.00 | (0.02) | 0.50 | (0.17) |
|  |  | | **Pers** | **Up** | 0.00 | (0.00) | 0.09 | (0.19) | 0.69 | (0.22) |
|  |  | |  | **Down** | 0.02 | (0.05) | 0.00 | (0.00) | 0.70 | (0.13) |
| **Total** | **Female** | | **Sex** | **Up** | 0.00 | (0.02) | 0.05 | (0.11) | 0.64 | (0.24) |
|  |  | |  | **Down** | 0.03 | (0.07) | 0.00 | (0.01) | 0.67 | (0.20) |
|  |  | | **Pers** | **Up** | 0.00 | (0.00) | 0.10 | (0.17) | 0.71 | (0.26) |
|  |  | |  | **Down** | 0.02 | (0.04) | 0.01 | (0.05) | 0.72 | (0.21) |
|  | **Male** | | **Sex** | **Up** | 0.00 | (0.01) | 0.09 | (0.17) | 0.52 | (0.20) |
|  |  | |  | **Down** | 0.03 | (0.06) | 0.00 | (0.01) | 0.53 | (0.16) |
|  |  | | **Pers** | **Up** | 0.00 | (0.00) | 0.12 | (0.20) | 0.65 | (0.20) |
|  |  | |  | **Down** | 0.03 | (0.05) | 0.01 | (0.03) | 0.73 | (0.14) |

Note. Mean and standard deviation (in brackets) for the number of fixations split by AOIs, condition (sexualized personalized), orientation (up, down), gender of the picture (male, female) and gender of the participant (male, female).

**Supplementary methods**

Participants of Experiment 1

One hundred forty-six healthy students (*N* = 87 men and *N* = 59 women; age *M* = 23.93, *SD* = 3.7 years) took part in the present study in exchange for monetary reward. The study was conducted at the SISSA. All participants gave written informed consent before participating in the study, which was approved by the SISSA ethical committee and were treated in accordance with the Declaration of Helsinki. Participants were naïve to the aim of the study and had not participated in similar studies before.

Procedure of Experiment 1

Participants took part in a picture recognition task with a similar procedure as in Bernard at al. (2012), but with a novel set of pictures and in a between-subjects design. Participants were randomly assigned to one of the three experimental groups: sexualized condition (*N* = 28 men and *N* = 19 women), personalized condition (*N* = 28 men and *N* = 20 women), and mannequin condition (*N* = 31 men and *N* = 20 women). Each group was presented with a total number of 48 pictures: 24 target pictures (personalized women, sexualized women or mannequins, depending on the experimental group) and 24 pictures of houses used as a control condition. Twelve pictures from each condition were presented in the upright orientation while the remaining 12 were inverted on the x axis (top-down). For each trial, a picture appeared in the middle of the computer screen for 250 *ms*, followed by a blank screen for 1000 *ms*. Immediately after, participants were presented with two pictures, one on the left side of the screen and one on the right, in which one of the two was the original picture, and the other was its left-right mirrored version. Participants were requested to indicate which one of the two pictures they had previously seen (S2 Fig) by pressing a key on the keyboard corresponding to the right picture (i.e., the “L” letter), or the left one (i.e., the “A” letter). Orientation and order of presentation were pseudo-randomized: with the orientation of the 24 images changing in two possible versions, while the order of presentation inside each version was always random. Pictures were presented on a computer screen using Cogent Toolbox (http://www.vislab.ucl.ac.uk/cogent.php), running on Matlab 2011a. Before starting the experiment, participants completed four practice trials, in order to familiarize with the task. The individual patterns of responses were recorded and analyzed both for women/mannequins and houses, in upright and inverted orientation.

Accuracy analyses were performed using RStudio software (version 3.3.2). As for the analyses on the mediating role of asymmetry for the SBIE, the bootstrap Lavaan R software [[38](#_ENREF_38)] was used with 1000 iterations for the implementation of a Structural Equation model without latent variables. The analyses on asymmetry and pretest evaluations were instead performed using IBM statistics software SPSS, version 21.

**Supplementary results**

***Analysis of the pretest of Experiments 1 and 2***

Participants’ ratings were analyzed by a one-way ANOVA with the between participant factors group (personalized, personalized and mannequin) and gender of the participant (male, female) separately for the Sexiness, Attractiveness, Intelligence and Familiarity dimensions as shown in Table S1.

Participants’ ratings on the Sexiness dimension were affected by the factor group, *F* (2, 69) = 448.71, *p* < .001, *η_p_^2^* = .93. Sexualized pictures were rated as sexier than the personalized ones (*p* < .001), and the mannequins (*p* < .001); the personalized pictures were rated as sexier than the mannequins (*p* < .001). A main effect of the gender of the participants was significant, *F* (1, 69) = 42.38, *p* < .001, *η_p_^2^* = .38, with the female participants rating the pictures as sexier than the male participants. A significant interaction of group and gender of the participant was also significant, *F* (2, 69) = 28.72, *p* < .001, *η_p_^2^* = .45, with male participants rating both the sexualized and personalized pictures as sexier as compared to the female participants (*p* < .001). The female participants rated the mannequins as sexier than the male participants (*p* = .04).

Participants’ ratings on the Attractiveness dimension were affected by the group variable, *F* (2, 69) = 415.08, *p* < .001, *η_p_^2^* = .92. Sexualized pictures were rated as more attractive than the personalized ones (*p* = .04), and the mannequins (*p* < .001); the personalized pictures were rated as more attractive than the mannequins (*p* < .001). A main effect of gender of the participants was significant, *F* (1, 69) = 23.27, *p* < .001, *η_p_^2^* = .25, with the male participants rating the pictures as more attractive than the female participants. A significant interaction of group and gender of the participant was also significant, *F* (2, 69) = 24.03, *p* < .001, *η_p_^2^* = .41, with male participants rating both the sexualized and personalized pictures as more attractive as compared to the female participants (*p* < .001). The female participants rated the mannequins pictures as more attractive than the male participants (*p* = .01).

Participants’ ratings on the Intelligence dimension were affected by the factor group, *F* (2, 69) = 324.25, *p* < .001, *η_p_^2^* = .90. Personalized pictures were rated as more intelligent than the sexualized pictures (*p* < .001), and the mannequins (*p* < .001). The sexualized pictures were rated as more intelligent than the mannequins (*p* < .001). A main effect of the gender of the participants was not significant, *F* (1, 69) = .667, *p* = .42, *η_p_^2^* = .01. However, a significant interaction of group and gender of the participant was found, *F* (2, 69) = 7.10, *p* = .002, *η_p_^2^* = .17, with male participants rating the sexualized pictures as more intelligent than the female participants (*p* = .02). The female participants rated the mannequin pictures as more intelligent than the male participants (*p* = .01). Personalized pictures were rated as similarly intelligent by female and male participants (*p* = .15).

Participants’ ratings on the Familiarity dimension were affected by the factor group, *F* (2, 69) = 38.66, *p* < .001, *η_p_^2^* = .53. Personalized pictures were rated as more familiar than the mannequins (*p* < .001), and the sexualized pictures (*p* < .001); no significant difference was found between sexualized and mannequins pictures (*p* = .10). Note that a separate analysis for pictures in the upright and inverted orientations was not necessary given that every picture was randomly assigned to both conditions. A main effect of gender of the participants was significant, *F* (1, 69) = 247.98, *p* < .001, *η_p_^2^* = .78, with the male participants rating the pictures as more familiar than the female participants. A significant interaction of group and gender of the participant was also significant, *F* (2, 69) = 24.03, *p* < .001, *η_p_^2^* = .41, indicating that female participants rated the sexualized pictures as more familiar than the mannequins pictures (*p* = .18), but the personalized pictures as more familiar than the sexualized ones *(p* < .001) and the mannequins. The male participants rated the personalized pictures as more familiar than both the sexualized (*p* = .01) and the mannequin pictures (*p* < .001), and the sexualized pictures as more familiar than the mannequins (*p* < .001).

***Analysis of the pretest Experiment 4***

Participants’ ratings were analyzed by a 2 (condition: sexualized, personalized) x 2 (gender of the picture: male, female) x 2 (gender of the participants: male, female) repeated measures ANOVA separately for the Sexiness, Attractiveness, Intelligence and Familiarity dimensions as shown in Table S2.

Participants’ ratings on the Sexiness dimension were affected by the factor gender of the participants, *F* (1, 92) = 13.93, *p* < .001, *η_p_^2^* = .13, meaning that pictures were rated as sexier by female participants than the male ones. A main effect of gender of the picture was found to be significant, *F* (1, 92) = 30.29, *p* < .001, *η_p_^2^* = .25, meaning that female pictures were rated as sexier than the male ones. A main effect of condition was found to be significant, *F* (1, 92) = 14.45, *p* < .001, *η_p_^2^* = .14, meaning that sexualized pictures were rated as sexier than the personalized one. The interaction of gender of the picture x condition was also found to be significant, *F* (1, 92) = 7.81, *p* = .01, *η_p_^2^* = .08, meaning that only the sexualized female pictures were rated as sexier than the personalized ones (*p* < .001) but the same was not true for the male pictures (*p* = .59). The other main effect and interaction did not approach the significance level *p* > .25.

Participants’ ratings on the Attractiveness dimension were affected by the factor gender of the participants *F* (1, 92) = 28.89, *p* < .001, *η_p_^2^* = .24 meaning that pictures were rated as more attractive by the female participants than the male one. A main effect of gender of the picture was found to be significant, *F* (1, 92) = 40.20, *p* < .001, *η_p_^2^* = .30, meaning that female pictures were rated as more attractive than the male ones. The other main effect and interaction did not approach the significance level *p* > .12.

Participants’ ratings on the Intelligence dimension were affected by the factor gender of the participant, *F* (1, 92) = 83.33, *p* < .001, *η_p_^2^* = .48, meaning that pictures were rated as more intelligent by the female participants than the male ones. A main effect of condition was found to be significant, *F* (1, 92) = 29.08, *p* < .001, *η_p_^2^* = .24, meaning that personalized pictures were rated as more intelligent than the sexualized ones. The other main effect and interaction did not approach the significance level *p* > .08.

Participants’ ratings on the Familiarity dimension were affected by the factor gender of the picture, *F* (1, 92) = 20.21, *p* < .001, *η_p_^2^* = .18, meaning that female pictures were rated as more familiar than the male ones. The other main effect and interaction did not approach the significance level *p* > .16.

***Similarity analysis of the pretest scores between the Experiments 1 - 2 and Experiment 3.***

The different asymmetries of the pictures between Experiments 1 and 2 and Experiment 3 could also impact the way in which the pictures are perceived in terms of the Sexiness, Attractiveness, Intelligence and Familiarity dimensions. A separate analysis was conducted to compare the four dimensions analyzed in the pretest among the set of female stimuli used in the Experiments 1 and 2 and Experiment 3.

Participants’ ratings were analyzed by a 2 (condition: sexualized, personalized) x 2 (Experiment: Experiment1-2, Experiment 3) x 2 (gender of the participant: male, female) univariate ANOVA separately for the Sexiness, Attractiveness, Intelligence and Familiarity dimensions. Since each of the stimuli sets has already been analyzed separately we here report only the results on the three-way interaction (condition x Experiment x gender of the participant) as it best addresses our research question.

The interaction of condition x Experiment x gender of the participants was found not significant for the Sexiness, *F* (1, 184) = .17, *p* = .68, *η_p_^2^* = .001, the Attractiveness (1, 184) = 1.83, *p* = .18, *η_p_^2^* = .01, the Intelligence (1, 184) = 1.33, *p* = .25, *η_p_^2^* = .01, and the Familiarity dimension (1, 184) = 1.74, *p* = .19, *η_p_^2^* = .01.

These results show how, despite the changed symmetrical features, the two stimuli datasets are still perceived similar in terms of Sexy, Attractive, Intelligent and Familiar dimensions.

#### *Analysis of the accuracy scores of Experiment 1*

Individual accuracy in the matching task was analyzed with the same settings described in Experiment 2, using a *generalized linear mixed effect* model (*glmer* R package) with an independent random intercept for every subject and with condition (houses, sexualized, personalized, mannequin), orientation (upright, inverted) and gender of the participant (male, female) as fixed effects.

In order to optimize the statistical validity of our dataset we decided to exclude from the analysis participants collecting a performance below the chance level (i.e., 75% correct) in more than 37.5% of tested experimental conditions (n= 4). This results in the exclusion of 29 participants and a final number of 117 participants (*N* = 77 men and *N* = 40 women). After the application of this exclusion criteria, we performed an outlier analysis on individual pattern of correct/ incorrect responses: trials in which any one of the considered individual binary response deviated more than 4 SD from the individual best fitting *glmer* model, including all interaction terms as fixed factors, were removed from the analyses, no trials were removed from the analyses (5616 trials in total) (for a similar outlier analysis see Ratcliff [[45](#_ENREF_45)]; Piccoli et al., [[46](#_ENREF_46)]).

We used type 3-like two tailed p-values for significance estimates of *glmer*’s fixed effects and parameters adjusting for the F-tests the denominator degrees-of freedom with the Satterthwaite approximation based on SAS proc mixed theory [[47](#_ENREF_47)].

The analysis based on the *glmer* model revealed a significant main effect of the gender of the participant, with men having/performing with a higher accuracy than women (*glmer* estimated accuracy for men vs. women = .93 ± .011 vs. .90 ± .017, *z* = 38.06, *p* <. 001; *F* (1, 5615) = 10.09, *p* = .001). The gender however did not significantly interact with any other variable *p* >.19. A main effect of the orientation was found, *F* (1, 5615) = 6.472, *p* = .01, which was further qualified by the interaction with the condition, *F* (2, 5615) = 6.19, *p* < .001. Note that the same orientation by condition interaction was also investigated separately (in a new *glmer* model containing only the orientation and condition as fixed effect and the participants as random effects) for the mannequin group *F* (1, 2016) = 9.61, *p* = .002; for the personalized group *F* (1, 1584) = 2.33, *p* = .13, and the sexualized group *F* (1, 2016) = 5.05, *p* = .02. In order to clarify the origin of such an interaction we looked at the effect of orientation on the accuracy, separately for each condition. To do so we ran *glmer* models containing only the orientation as the main effect and the participants as random effect, separately for each condition subset. Analyses revealed that, pictures were better recognized in the upright than the inverted orientation in the personalized (*glmer* estimated accuracy for upright vs. inverted = .96 ± .024 vs. .90 ± .04, *z* = 3.13, *p* =. 002; *F* (1,791) = 9.81, *p* = .001) and in the mannequin condition (*glmer* estimated accuracy for upright vs. inverted = .96 ± .020 vs. .91 ± .03, *z* = 3.35, *p* <. 001; *F* (1, 1007) = 11.17, *p* < .001) but not in the sexualized condition (*glmer* estimated accuracy for upright vs. inverted = .96 ± .019 vs. .94 ± .024, *z* = 1.83, *p* = . 07; *F* (1, 1007) = 3.35, *p* = .07).

Notably the pattern of matching accuracy resulting from the sexualized condition was similar to the one resulting from the house (baseline) condition with upright and inverted houses (*glmer* estimated accuracy for upright vs. inverted = .90 ± .02 vs. .91 ± .02, *z* = .832, *p* = .41; *F* (1, 2808) = .69, *p* = .51). In addition, to gather evidence that houses were equally well recognized in the upright and inverted orientation and, most importantly for our purpose, that the recognition of the houses was similar for the three experimental groups, we also run a *glmer* model containing only the orientation as the main effect and the participants as random effects, separately for the houses subsets of each group. Results revealed that houses were recognized with a similar accuracy rate in the upright and the inverted orientation in the sexualized (*glmer* estimated accuracy for upright vs. inverted = .91 ± .03 vs. .91 ± .03, *z* =1.02, *p* =. 31; *F* (1, 1007) = 1.74, *p* = .19), in the personalized (*glmer* estimated accuracy for upright vs. inverted = .87 ± .03 vs. .90 ± .03, *z* = 14.34, *p* <. 001, *F* (1, 1007) = 1.04, *p* = .30) as well as in the mannequin condition (*glmer* estimated accuracy for upright vs. inverted = .89 ± .03 vs. .91 ± .03, *z* =14.89, *p* <. 001; *F* (1, 1007) = .73, *p* = .39). Hence, this allowed us to rely on the accuracy score of houses as a proper baseline condition (See Fig S3).

#### *Mediation analyses on the asymmetry index of Experiment 1*

In order to ascertain whether the SBIE was mainly driven by the different asymmetry between the condition stimuli, a mediation analysis was conducted with the same parameter described in Experiment 2 reported in the main text.

The analysis revealed a good fit of the model [[42](#_ENREF_42)] with the Comparative Fit Index (CFI) always larger than 0.95 (CFI = 0.98, 1st to 3rd quartile 0.992 to 0.999) and the Standardized Root Mean-square Residual (SRMR) always smaller than 0.08 (SRMR = 0.037, 1st to 3rd quartile 0.018 to 0.045). The model was characterized by a significant Total Effect (*c* path) (*r^2^* = 0.162, *F*_3, 92_ = 5.929, *p* < 0.001) with a minimal though not different from zero inversion effect for the house condition (*lm* estimated inversion effect = -0.0129 ± 0.0147, *t* = -0.880, *df* = 92, *p* = 0.381), which was smaller than the inversion effect found in both the mannequin (*lm* estimated inversion effect difference from the house inversion effect = 0.0747 ± 0.0207, *t* = 3.603, *df* = 92, *p* < 0.001) and the personalized women condition (*lm* estimated inversion effect difference from the house inversion effect = 0.074 ± 0.02073, *t* = 3.589, *df* = 92, *p* < 0.001), but not from the one found in the sexualized women condition (*lm* estimated inversion effect difference from the house inversion effect = 0.037 ± 0.0207, *t* = 1.79, *df* = 92, *p* = 0.08). Notably the magnitude of the Total Effect calculated on the inversion effect values was of about the same statistical entity of the condition × orientation interaction revealed by the *glm* analysis on the individual pattern of correct responses. This showed that the 96 inversion effect values used here to infer the mediating role of asymmetry on the performance provided a reliable synthetic measure of individual performance.

Furthermore, as the condition significantly contributed to the variance of the inversion effect, it also contributed to the variance of the asymmetry (SEM estimated coefficient = 5.822 ± 0.794; *z* = 7.331, *p* < 0.001; *r^2^* = 0.58, *F*_3, 92_ = 41.67, *p* < 0.001), with the asymmetry of the mannequin (*lm* estimated asymmetry = 8.96 ± 2.079, *t* = 4.313, *df* = 92, p <0.001) and of the personalized women (*lm* estimated asymmetry = 14.91 ± 2.079, *t* = 7.172, *df* = 92, p < 0.001), been intermediate and the one of the sexualized women (*lm* estimated asymmetry = 22.45 ± 2.079, *t* = 10.797, *df* = 92, *p* < 0.001) been maximal relative to the asymmetry imposed in the present analysis to houses as a baseline (i.e., 0). This result corroborated the condition by asymmetry co-variation revealed by the preliminary analysis of the asymmetry of the stimuli used in our dataset.

The relation between asymmetry and inversion effect (*b* path) resulted to be only partially reliable (SEM estimated coefficient = -0.002 ± 0.001, *z* = -2.293, *p* = 0.02), being not significant when considered directly (*r^2^*= 0.0002, *F*_1,94_ = 0.017, *p* = 0.89; *lm* estimated coefficient = -0.0009 ± 0.00073, *t* = -0.133, *df* = 92, *p*= 0.89) following James and Brett [[48](#_ENREF_48)], and significant when considered indirectly following Baron and Kenny [[35](#_ENREF_35)] as controlling for the effect of the condition as a causal variable (*lm* estimated coefficient = -0.002, ± 0.001, *t* = -2.498, *df* = 91, p < 0.001). The low reliability of such a mutual relationship only partially fulfills the criteria for the establishment of an asymmetry mediation of the total effect. However, the lack of mediation is fully demonstrated by the fact that the direct association between condition and inversion effect was not significantly affected by the addition of the asymmetry as a mediator (*c*’ path) (SEM estimated coefficient = 0.030 ± 0.008, *z* = 3.192, *p* = 0.001; *F*_3,91_ = 6.242, *p* = 0.01). This was further corroborated by the fact that a significant loss in the fit was found when contrasting a *lm* model with asymmetry as the only predictor of inversion effect vs. a *lme* model including both asymmetry and condition (*F* = 8.34, *df* = 3, *p* < 0.001). The results of the mediation analysis thus provide no evidence that the asymmetry of the images mediates the differential effect of inversion on matching performance observed among the different categories of images.

***On the moderating role of asymmetry in Experiment 4***

Although the asymmetry of the pictures was kept equal among conditions and gender of the pictures, their distribution is still continuous. Therefore, we run a *glmer* model containing in addition to the factors already described for the accuracy analyses of the Experiment 4 (i.e. condition (sexualized, personalized) x orientation (upright, inverted) x gender of the picture (male, female)), an additional fixed factor: asymmetry (low, high). Pictures were categorized as low and high asymmetrical according to a two median split of their asymmetry index. The analyses revealed a significant main effect of the asymmetry indicating that the high asymmetrical pictures were better recognized than the low asymmetrical ones (*glmer* estimated accuracy for high vs. low = .94 ± .01 vs. .91 ± .02, *z* = 3.24, *p* < .001; F (1, 4992) = 25.20, *p* < .001). A main effect of orientation was also found indicating that pictures were better recognized in the upright than in the inverted orientation (*glmer* estimated accuracy for upright vs. inverted = .93 ± .01 vs. .91 ± .02, *z* = 2.00, *p* = .05; *F* (1, 4992) = 4.07, *p* = .04). However, given the complexity of the model, we run a *glmer* model containing only the orientation as the main effect and the participants as random effect, separately for each condition and asymmetry level subset. Analyses (1 tailed) revealed that only in the personalized condition the low asymmetrical pictures were better recognized in the upright than in the inverted orientation (*glmer* estimated accuracy for upright vs. inverted = .94 ± .02 vs. .90 ± .03, *z* = 1.77, *p* = .04; *F* (1, 1404) = 3.14, *p* = .04), while in the sexualized condition they were recognized equally well in the two orientations (*glmer* estimated accuracy for upright vs. inverted = .91 ± .03 vs. .88 ± .03, *z* = 1.25, *p* = .11; *F* (1, 1092) = 1.57, *p* = .11). On the contrary high asymmetrical pictures were recognized equally well in the two orientations in both the personalized (*glmer* estimated accuracy for upright vs. inverted = .94 ± .02 vs. .93 ± .02, *z* = 1.17, *p* = .12; *F* (1, 1092) = 1.37, *p* = .12) and the sexualized condition (*glmer* estimated accuracy for upright vs. inverted = .94 ± .02 vs. .95 ± .02, *z* = .42, *p* = .34; *F* (1, 1404) = .18, *p* = .34).

***Full analysis of the eye movements: fixation duration (FD)***

A 2 condition (sexualized, personalized) x 2 orientation (upright, inverted) x 2 gender of the picture (male, female) x 2 gender of the participant (male, female) within-subjects ANOVA was carried out on eye movement data (mean fixation duration and total number of fixations) separately for each AOI (see Fig 6 and Table S6 and S7).

***Face AOI***

A main effect of gender of the picture was found, F (1, 50) = 6.76, p = .01, *η_p_^2^* = .12, meaning that the Face AOI of the male pictures was fixated longer than the female ones. A main effect of condition was found, F (1, 50) = 7.81, *p* = .01, *η_p_^2^* = .14, meaning that the Face AOI of the personalized pictures was fixated longer than the sexualized ones. A main effect of orientation was found, F (1, 50) = 18.70, p < .001, *η_p_^2^* = .27, meaning that the Face AOI of the upright pictures was fixated longer than the inverted ones. An interaction of gender of the picture x orientation was found, *F* (1, 50) = 6.84, *p* = .01, *η_p_^2^* = .12, meaning that when presented upright, male pictures were fixated in the Face AOI longer than female pictures (*p* = .01). The Face AOI of the pictures presented inverted was equally fixated in the female pictures and the male ones (*p* = .97). An interaction of condition x orientation was also found, *F* (1, 50) = 5.98, *p* = .02, *η_p_^2^* = .12, meaning that the personalized pictures were fixated longer in the Face AOI than the sexualized pictures only when presented in the upright orientation (*p* = .01) as compared to the inverted one (*p* = .10). An interaction of condition x orientation x gender of the participant x gender of the picture was also found, *F* (1, 50) = 4.00, *p* = .05, *η_p_^2^* = .07, meaning that female participants fixated the Face AOI of the female sexualized pictures equally longer in the two orientations (*p* = .10) as compared to the other conditions where the face of the upright pictures was fixated longer than of the inverted ones (*p* < .06). All the other effects and interactions did not approach the significance level (*p* > .10).

***Breast AOI***

A main effect of gender of the picture was found, *F* (1, 50) = 29.54, *p* < .001, *η_p_^2^* = .37, meaning that the Breast AOI of female pictures was fixated longer than the male ones. A main effect of condition was found, *F* (1, 50) = 77.16, *p* < .001, *η_p_^2^* = .61, meaning that the Breast AOI of the personalized pictures was fixated longer than the sexualized ones. A main effect of the orientation was found *F* (1, 50) = 17.06, *p* < .001, *η_p_^2^* = .25 meaning that the Breast AOI of the inverted pictures was fixated longer than the upright ones. An interaction of orientation x gender of the participant was found, *F* (1, 50) = 4.88, *p* = .03, *η_p_^2^* = .09, meaning that only female participants fixated the Breast AOI of the inverted pictures longer than the upright ones (*p* < .001) (*p* = .02). An interaction of condition x gender of the picture was found, *F* (1, 50) = 19.22, *p* < .001, *η_p_^2^* = .28, meaning that only in the sexualized condition the Breast AOI of the female pictures was fixated longer than the male pictures (*p* < .001). All the other effects and interactions did not approach the significance level (*p* > .08).

***Pelvic AOI***

A main effect of orientation was found *F* (1, 50) = 14.24, *p* < .001, *η_p_^2^* = .25, meaning that the Pelvic AOI of the inverted pictures was fixated longer than the upright ones. All the other effects and interactions did not approach the significance level *p* > .16.

***Full analysis of the eye movements: number of fixations (NF)***

***Face AOI***

A main effect of gender of the picture was found, F (1, 50) = 7.21, p = .01, *η_p_^2^* = .13, meaning that the Face AOI of the male pictures was fixated more times than the female ones. A main effect of condition was found, F (1, 50) = 13.90, p < .001, *η_p_^2^* = .22, meaning that the Face AOI of the personalized pictures was fixated more times than the sexualized ones. A main effect of orientation was found, F (1, 50) = 18.91, p < .001, *η_p_^2^* = .27, meaning that the Face AOI of the upright pictures was fixated more times than the inverted ones. An interaction of gender of the picture x orientation was found, *F* (1, 50) = 4.85, *p* = .03, *η_p_^2^* = .09, meaning that when presented upright, male pictures were fixated in the Face AOI more times than female pictures (*p* = .01). The Face AOI of the pictures presented inverted was fixated similarly often in the female pictures and the male ones (*p* = .85). An interaction of condition x orientation was also found, *F* (1, 50) = 8.59, *p* = .01, *η_p_^2^* = .15, meaning that the personalized pictures were fixated more times in the Face AOI than the sexualized pictures only when presented in the upright orientation (*p* = .001) as compared to the inverted one (*p* = .06). An interaction of condition x orientation x gender of the participant x gender of the picture was also found, *F* (1, 50) = 3.93, *p* = .05, *η_p_^2^* = .07, meaning that male participants fixated the Face AOI of the sexualized male pictures an equal amount of times in the two orientations (*p* = .12) as compared to the other conditions in which the face of the upright images was always fixated more than times than of that inverted ones (*p* < .03).

All the other effects and interactions did not approach the significance level (*p* > .06).

***Breast AOI***

A main effect of gender of the picture was found, *F* (1, 50) = 28.00, *p* < .001, *η_p_^2^* = .36, meaning that the Breast AOI of female pictures was fixated more times than the male ones. A main effect of condition was found, *F* (1, 50) = 60.84, *p* < .001, *η_p_^2^* = .55, meaning that the Breast AOI of the personalized pictures was fixated more often than the sexualized ones. An interaction of orientation x gender of the participant was found, *F* (1, 50) = 6.29, *p* = .02, *η_p_^2^* = .11, meaning that only female participants fixated the Breast AOI of the inverted pictures more times than the upright ones (*p* = .02). A interaction of condition x gender of the picture was found, *F* (1, 50) = 22.90, *p* < .001, *η_p_^2^* = .31, meaning that only in the sexualized condition the Breast AOI of the female pictures was fixated more times than the male pictures (*p* < .001). A trend for the interaction of condition x gender of the picture x gender of the participant was found, *F* (1, 50) = 3.37, *p* = .07, *η_p_^2^* = .06, meaning that female participants fixated the Breast AOI of the sexualized and personalized female pictures equally often (*p* = .22), whereas male participants fixated the Breast AOI of the personalized pictures more times than of the sexualized pictures (*p* = .004). All the other effects and interactions did not approach the significance level (*p* > .10).

***Pelvic AOI***

A main effect of orientation was found, *F* (1, 50) = 15.66, *p* < .001, *η_p_^2^* = .24, meaning that the Pelvic AOI of the inverted pictures was fixated more times than in the upright ones.
